# USP13 promotes enzalutamide resistance by catalyzing depolyubiquitination of PCMT1 in prostate cancer

**DOI:** 10.1038/s41419-026-08824-9

**Published:** 2026-04-30

**Authors:** Zhipeng Wang, Xiaoqiang Liu, Zhongqi Li, Ruize Yuan, Fuchun Zheng, Situ Xiong, Jin Zeng, Wan Pang, Bin Fu, Sheng Li, Songhui Xu, Jun Deng

**Affiliations:** 1 Department of Urology, The First Affiliated Hospital, Jiangxi Medical College, Nanchang University, Nanchang, China; 2 Jiangxi Institute of Urology, Nanchang, China; 3 Department of Urology, The Second Hospital of Hebei Medical University, Shijiazhuang, China; 4 College of Chemical and Environmental Engineering, Shanghai Institute of Technology, Shanghai, China

**Keywords:** Biochemistry, Cell biology

## Abstract

Castrate resistant prostate cancer (CRPC) is often driven by constitutively active androgen receptor and AR splicing variants that become resistant to established hormonal therapy strategies such as enzalutamide. Deubiquitinating enzymes (DUBs) play crucial roles in cancer development, progression, and metastasis by epigenetic modification. Hence, targeting DUBs might prove to be a valid strategy for developing novel anti-cancer therapeutics. Here, we reveal that the deubiquitinating enzyme USP13 is up-regulated in PCa tissues and correlates with prostate cancer progression. USP13 silencing inhibits prostate cancer cell growth in vitro and in vivo. Mechanically, USP13 directly interacts with PCMT1 and removes polyubiquitination of PCMT1 to maintain its stability, which promotes PCa cell proliferation and enzalutamide resistance. Depletion of USP13 promoted PCa cells sensitive to enzalutamide. Clinically, USP13 was significantly up-regulated in prostate cancer tissues and positively associated with PCMT1 expression. Notably, inhibition of USP13 significantly decreases prostate tumor growth and improves enzalutamide treatments through PCMT1 suppression. Our studies demonstrate that inhibition of USP13 can offer a viable therapeutic option to overcome enzalutamide resistance in prostate cancer patients with USP13/PCMT1-overexpression.

## Introduction

Enzalutamide, a second-generation androgen receptor (AR) antagonist, which blocks AR activity, is the primary treatment for castration-resistant prostate cancer [1]. Unfortunately, patients receiving enzalutamide treatment will ultimately develop resistance via various complicated mechanisms. Therefore, identifying approaches to antagonize the enzalutamide resistance mechanism is critical for the development of effective treatments for castration-resistant prostate cancer.

Unlike ubiquitinating enzymes, the deubiquitinating enzymes (DUBs) are isopeptidases that cleave ubiquitin, either from the protein substrate itself or between ubiquitin moieties on polyubiquitin chains [2]. This cleavage can rescue proteins from degradation, alter signaling, and restore the pool of free ubiquitin in the cells [3]. Recent studies have shown that DUBs are critical in the development of drug resistance in many cancers [4]. USP13, a critical DUB, plays key roles in various physiological and pathological processes, including cancer metastasis [5], cancer stem [6], DNA damage [7], mitochondrial homeostasis [8], autophagy [9] and cell cycle [10] by regulating various substrates, including MCL1, SKP2, MITF, PTEN, TOPBP1, and c-MYC [11–16]. However, the roles of USP13 in different tumor types are controversial. USP13 functions as a tumor suppressor in oral squamous cell carcinoma, breast cancer, and bladder cancer by stabilizing and increasing the expression of PTEN [12, 17, 18]. While USP13 can function as a tumor promoter in other tumors, including kidney cancer, melanoma, ovarian cancer, lung cancer, and cervical cancer, by respectively regulating ZHX2 [19], MITF [14], and MCL1 [11, 20]. However, whether USP13 regulates enzalutamide resistance in prostate cancer remains unclear.

PCMT1, a repair enzyme, catalyzes the conversion of isomerized aspartic acid (iso-Asp) residues into their normal structure, thereby restoring the configuration and function of proteins [21]. PCMT1 has been reported to regulate the proliferation, apoptosis, and migration of different cancer cells, which further promotes the occurrence and development of cancers [22]. In lung adenocarcinoma, high expression of PCMT1 was related to invasive adenocarcinoma, and patients with high PCMT1 expression have a shorter survival. In bladder cancer, up-regulation of PCMT1 is closely related to clinical grade, muscle infiltration, lymph node metastasis, and distant metastasis of bladder cancer patients [23]. Recent studies showed that PCMT1 expression was significantly increased in prostate cancer, which promoted the proliferation, migration, and invasion of PCa cells by PI3K/AKT/GSK-3β signaling pathway [21]. But the molecular mechanism governing PCMT1 protein up-regulation in human cancers is still unclear. Therefore, the identification of the upstream factors regulating PCMT1 expression is urgent for demonstrating the biological function of PCMT1 and for potential prostate therapeutic interventions.

In this study, we revealed that upregulation of USP13 promoted PCa cell development and enzalutamide resistance. At the mechanistic level, USP13 binds and deubiquitins PCMT1, which enhances its stability. High USP13 expression showed a positive correlation with high PCMT1 levels in PCa tissues, implying that USP13 acts as a tumor promoter in prostate cancer. Targeting USP13 by Spautin-1 presents a promising approach to inhibit enzalutamide-resistant PCa.

## Materials and methods

### Cell lines and cell culture

HEK293T, C4-2B, and DU145 cells were purchased from the American Type Culture Collection(ATCC). All cell lines were cultured in RPMI-1640 or DMEM medium supplemented with 10% fetal bovine serum (FBS), 100 U/mL penicillin, and 0.1 mg/mL streptomycin. Routine testing for mycoplasma contamination was performed using PCR, and cell line authentication was conducted by short tandem repeat (STR) profiling. Authentication and quality control were carried out by the National Infrastructure of Cell Line Resources of China.

### Histopathology

Mouse prostate tissues were fixed overnight in 10% neutral buffered formalin, processed according to standard procedures, and embedded in paraffin. Paraffin-embedded tissues were cut into 5-micron-thick sections, dewaxed, rehydrated, and stained for Ki-67. Histological analysis of Ki-67-stained prostate tissue was performed by a board-certified pathologist. Cell proliferation index was calculated as the percentage of Ki-67 positive nuclei in total nuclei.

### Immunohistochemistry (IHC)

Prostate tissue microarray was purchased from Spector Biotech Inc. (Shandong, China) (Number: SP159) and the detailed information, including stage, age, classification, and so on in the Supplementary Tables S1 and S3. Clinic tissue sample collection was approved by the Internal Review and Ethics Boards of the First Affiliated Hospital of Nanchang University (Registration number: (2024) CDYFYYLK(10-026)). For immunohistochemical staining, paraffin-embedded prostate tissue sections were dewaxed, rehydrated, washed with gradient ethanol and PBS, and antigen retrieved using citrate buffer. Endogenous peroxidase activity was blocked with TBS/H_2_O_2_, and the primary antibody was incubated overnight at 4 °C. The next day, after 30 min of rewarming, incubation with the secondary antibody for another 30 min was followed by final staining with DAB and counterstaining with hematoxylin. USP13 and PCMT1 expression was scored semiquantitatively: 0 (no staining), 1 (weak), 2 (moderate), and 3 (strong), with scores of 2 and 3 indicating high expression. The average score from two pathologists established the final immunostaining score, and the chi-square (χ²) test was applied to statistically analyze USP13 and PCMT1 expression.

### Immunostaining

Slides containing cells were fixed in 4% paraformaldehyde for 15 min, washed, and immersed in PBS containing 0.5% Triton X-100 for 10 min. Block with serum for 30 min at room temperature and incubate overnight with primary antibody at 4 °C. The next day, the cells were incubated for 1 h with fluorescently labeled secondary antibody, and the nuclei were stained with DAPI. Observation was performed using a fluorescence microscope at ×400 or ×1000 magnification.

### Mouse tumor assay

All BALB/c nude mice (4 weeks of age) were from Charles River Laboratories in Beijing, China. Animals used in this study were humanely handled in accordance with applicable regulations, policies, and guidelines for animals. All experimental procedures using animals were approved by the Institutional Animal Care and Use Committee of the First Affiliated Hospital of Nanchang University (approval number: CDYFY-IACUC-202407QR186). For Fig. 2G–I, the indicated control C4-2B cells or USP13 knocked down C4-2B cells were subcutaneously injected into Balb/c nude mice. For Fig. 5E–G, C4–2B cells (1 × 10^6^) of the indicated Controls shUSP13 and shUSP13 + Flag-PCMT1 were mixed with Matrigel (1:1) and injected subcutaneously into the flanks of BALB/c nude male mice to complete the assay of mouse xenograft tumors. In order to establish the orthotopic model of prostate cancer in situ, the enzalutamide-resistant C4-2B cells of Control and shUSP13 were surgically injected into the prostate of mice after castration. The mice bearing enzalutamide-resistant C4-2B tumors and the USP13-silenced mice bearing enzalutamide-resistant C4-2B tumors were treated with vehicle control and USP13 inhibitor (10 mg/kg i.p.) two times per 5 days for 25 days. On 25 days, mice received 5 mg/kg D-fluorescein (YEASEN, 40902 ES 02) injection and were imaged 5 min later at the animal imaging facility of the First Affiliated Hospital of Nanchang University using an aLAGO imager and Aura imaging software (Spectral Instruments Imaging). The researchers analyzed the data by fluorescence intensity.

### Statistical analysis

Statistical analyses were performed with Prism 8.0 (GraphPad Software). All statistical comparisons were evaluated by the Student’s *t*-test or one-way or two-way analysis of variance (ANOVA). Among all the data sets, *p* values less than 0.05 were considered significant.

Additional methods are presented in the Supplemental Data.

## Results

### USP13 is up-regulated in prostate cancer tissues and positively correlates with prostate cancer progression

**Fig. 1:**
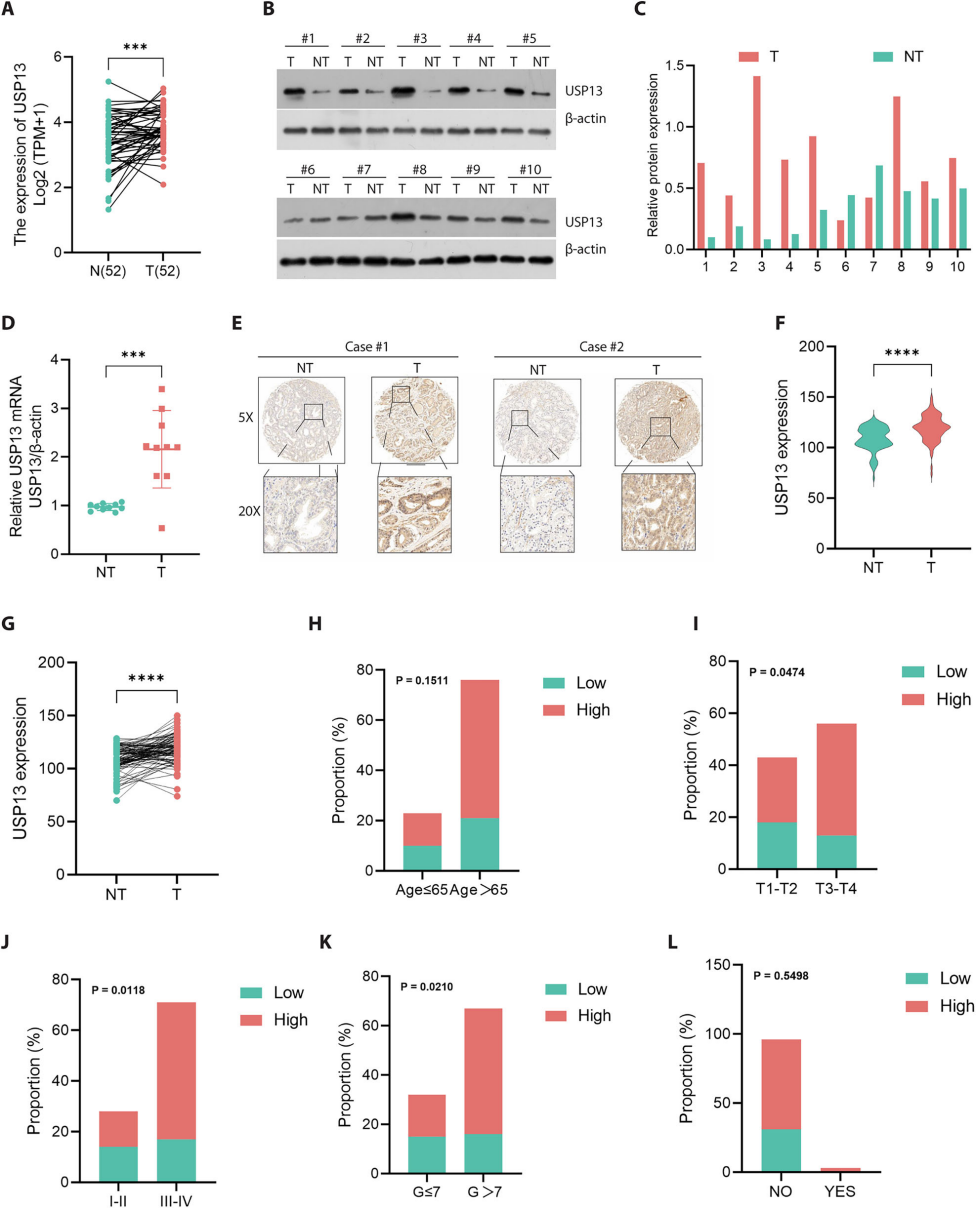
USP13 is up-regulated in prostate cancer tissues and positively correlates with prostate cancer progression. **A** USP13 mRNA was up-regulated in PCa tissues, which were analyzed from the TCGA database (https://portal.gdc.cancer.gov). Paired *t*-test, ^***^p < 0.001. **B**–**D** The protein expression and mRNA level of USP13 in adjacent non-tumor tissues (*n* = 10) and matched PCa tissues (*n* = 10) were respectively analyzed by Western Blot and qPCR. The software of Image J was used to quantify USP13 protein expression. Paired *t*-test, ^***^p < 0.001. **E** Representative immunohistochemical images of USP13 in PCa tissues and adjacent non-tumor tissues are shown. **F**, **G** The protein expression of USP13 was up-regulated in all PCa tissues (*n* = 99) compared with all adjacent non-tumor tissues (*n* = 99) and or matched adjacent non-tumor tissues. Paired *t*-test, ^****^p < 0.0001. **H**–**L** Overexpression of USP13 was not related to age (*p* = 0.01511) and tumor metastasis (*p* = 0.5498), but related to a higher preoperative clinical T3–T4 (*p* = 0.0474), Tissue stage III–IV (*p* = 0.01181), and Gleason score å 7 (*p* = 0.0210). Chi-square test was used.

To determine whether USP13 is up-regulated in prostate cancer, we first analyzed the data from TCGA. Figure 1A shows that the mRNA levels of USP13 were up-regulated in PCa tissues. To confirm this result, we next assessed USP3 expression in 10 adjacent non-tumor tissues and matched PCa tissues by Western Blot and Real-time PCR. As shown in Fig. 1B–D, both the USP13 protein and mRNA expression were up-regulated in PCa tissues. Next, we evaluated the USP13 expression in PCa tissues using USP13 IHC staining. We firstly validated the USP13 antibody by showing that it strongly stained PCa tissue section in contrast to negative staining of adjacent tissue section by control IgG (Fig. S1A). IHC was used to analyze USP1 expression in tissue microarray, which includes 100 samples of PCa tissues and 99 samples of adjacent non-tumor tissues (Fig. S1B). The IHC results (Fig. 1E-G) revealed that USP13 was up-regulated in PCa tissues compared with non-tumor tissues, and among the 99 PCa tissue samples, 68 cases had a strong USP13 expression while only 31 cases had a weak or negative USP13 expression (Table S1). Notably, overexpression of USP13 was positively correlated with larger tumor size and poor histological grade but not correlated with age and Lymph node metastasis (Fig. 1H–L and Table S1). These data indicated aberrant USP13 expression in prostate cancer, and elevated USP13 may be involved in PCa progression.

### USP13 promotes prostate tumor cell proliferation

**Fig. 2:**
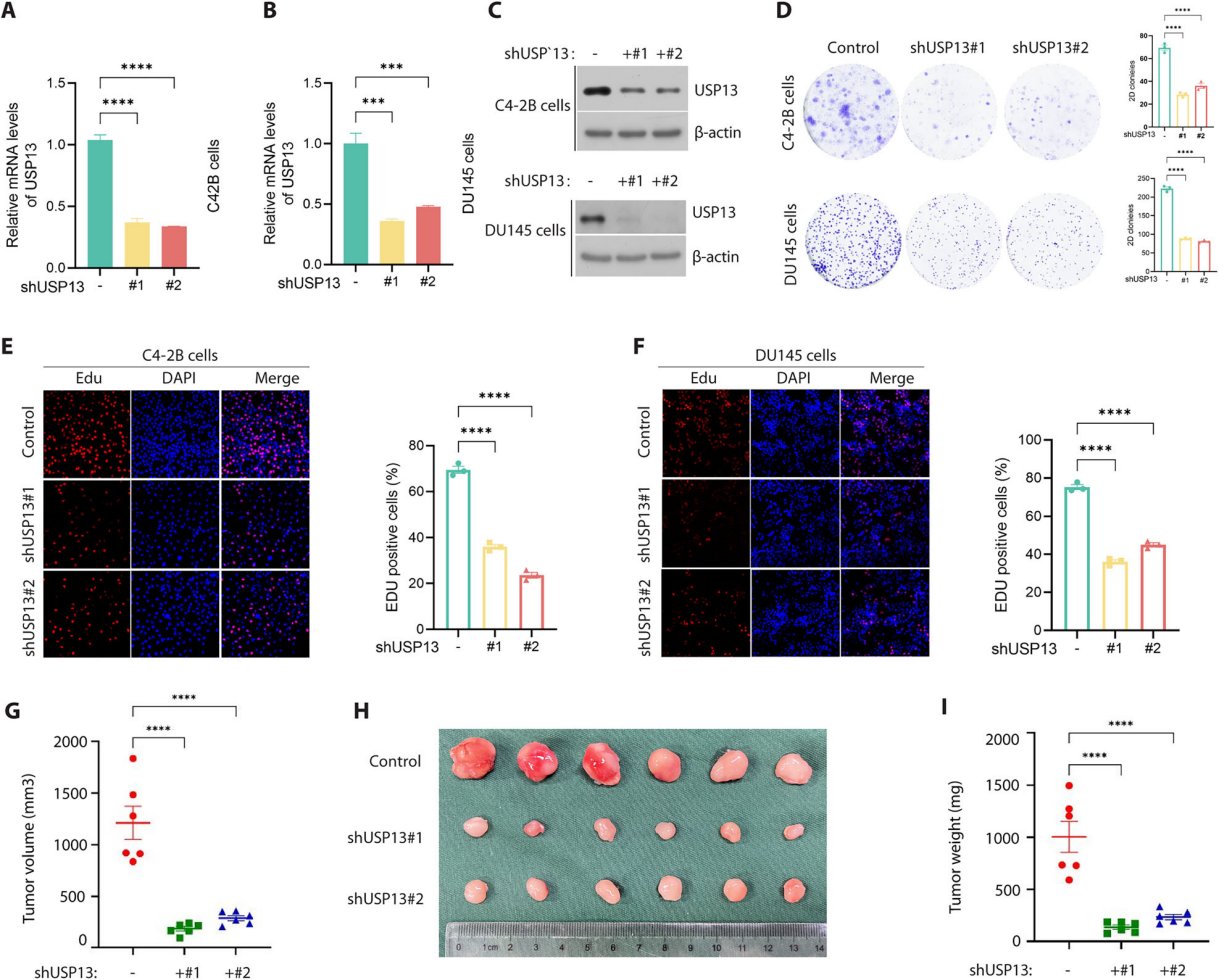
Knocking down of USP13 decreases PCa cell proliferation in vitro and vivo. **A**–**C** USP13 was knocked down in both C4-2B and DU145 cells by Lentiviral shRNA-mediated knockdown, which was confirmed by qPCR and Western Blot. Non-paired *t*-test, ^***^p < 0.001 and ^****^p < 0.0001. **D**–**F** The effects of the stable silencing of USP13 on C4-2B and DU145 cell colony formation (**D**) and proliferation (**E**, **F**) were determined. **D**, **E** Representative micrographs (left panel) and quantification (right panel) of Edu labeling in C4-2B and DU145 cells stably knocked down USP7. DAPI (4’,6-diamidino-2-phenylindole) staining was performed to visualize the nuclei. Non-paired *t*-test, ^***^p < 0.001 and ^****^p < 0.0001. **G**–**I** The indicated control C4-2B cells or USP13 knocked down C4-2B cells were subcutaneously injected into Balb/c nude mice. Tumor volume **G** was measured at 28 days (*n* = 5 per group). After 28 days, mice were sacrificed, and the tumor image (**H**) was presented, and tumor weights (**I**) were measured in the three groups. Statistical significance was determined using one-way ANOVA. ^****^p < 0.0001.

To determine the role of USP13 in prostate cancer, we first knocked down the expression of USP13 in both C4-2B and DU145 cells. The knocking down efficiency of USP13 was confirmed by Western Blot and Real-time PCR (Fig. 2A–C). Through colony formation assay and Edu assay, we assessed the influence of USP13 on tumor phenotype in both C4-2B and DU145 cells in vitro and found that USP13 knockdown reduced both prostate cancer cell proliferation (Fig. 2D–F). We also clarify whether USP13 is required for the survival and proliferation of non-cancerous prostate epithelial cells (RWPE-1) and castration-sensitive prostate cancer cells (LNCaP), and we found silencing of USP13 decreased these cells' colony formation (Fig. S2). To further determine the role of USP13 in vivo, we employed a prostate tumor model in which C4-2B cells were subcutaneously injected into nude mice. In control mice, injection of C4-2B cells resulted in the formation of large prostate tumors, while comparable injection of C4-2B cells transduced with either one of two different USP13 shRNAs indicated that USP13 knockdown significantly reduced tumorigenesis (Fig. 2G–I). Collectively, these data suggest that USP13 promotes long-term proliferation and survival of prostate cancer cells in vitro and in vivo.

### USP13 binds and interacts with PCMT1

**Fig. 3:**
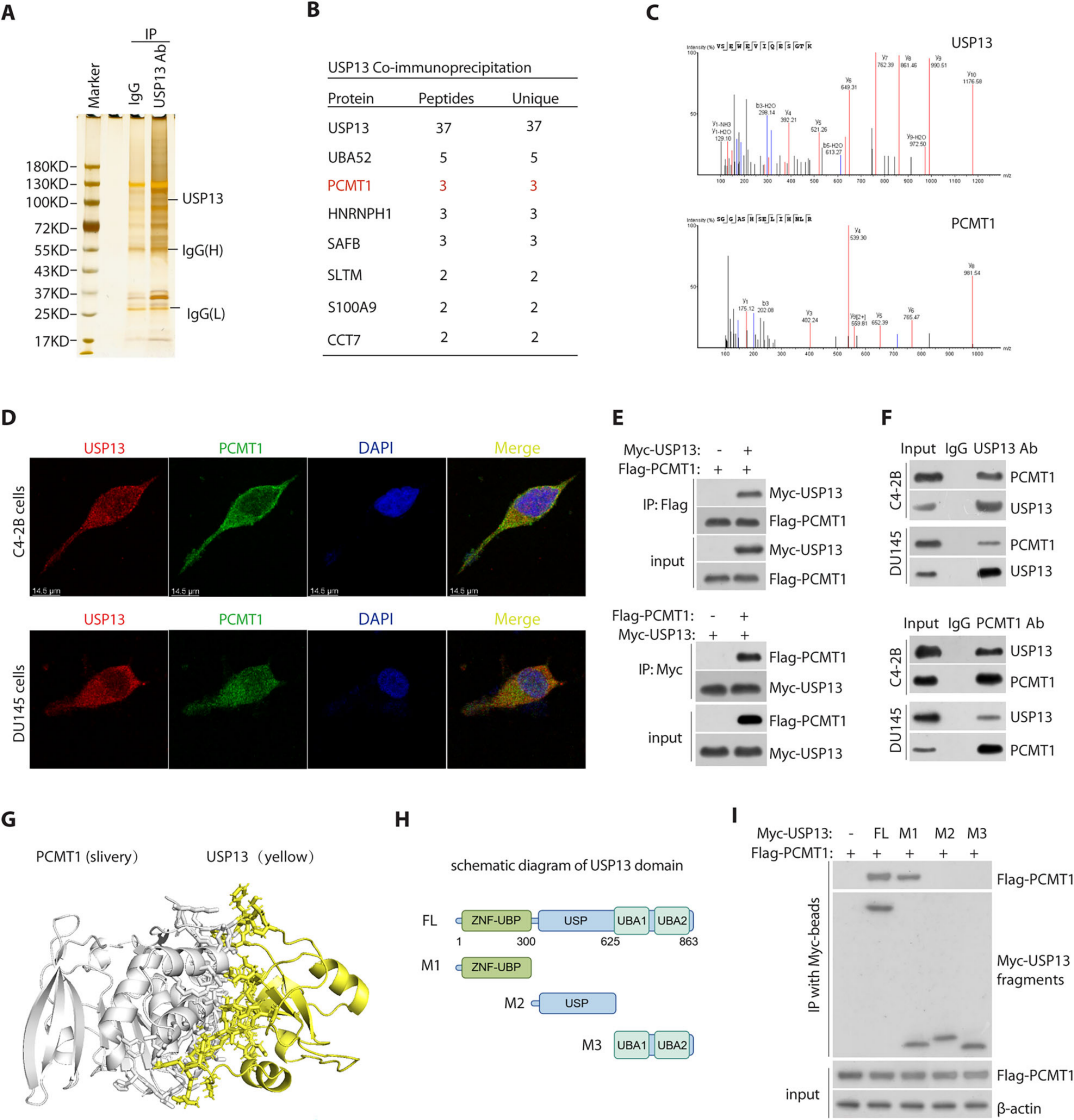
USP13 binds to PCMT1 and is associated with it. **A** Proteins that interacted with USP13 were identified by co-immunoprecipitation and silver staining. **B** List of USP13-associated proteins identified by mass spectrometric analysis (both total peptides and unique peptides ≥ 2). **C** Representative best unique peptides of USP13 and PCMT1 identified by mass spectrometry assays were exhibited. **D** Representative fluorescent images of PCMT1 (green) and USP13 (red) were immunostained using their antibody in C4-2B and DU145 cells. Nuclear 4’, 6-diamidino-2-phenylindole (DAPI; blue) was used to stain the nucleus. **E** HEK293T cells were co-transfected with Flag-PCMT1 and Myc-USP13, and cell lysates were subjected to IP with Flag-beads or Myc-beads and then detected with the indicated antibodies. **F** C4-2B cells or DU145 cells were subjected to immunoprecipitation with the indicated USP13 or PCMT1 antibodies and then detected by Western Blot with the corresponding antibodies. **G** The binding interface between USP13 and PCMT1 was based on the molecular docking model. The molecular docking analysis for the USP13-PCMT1 interaction was performed using the GRAMM web server (http://vakser.compbio.ku.edu/resources/gramm). **H** Diagram of the wild-type USP13 and mutation constructs with different domains. **I** HEK293T cells were co-transfected with Flag-PCMT1 and Myc-tagged full-length USP13 or its deletion mutants, and cell lysates were subjected to IP and detected with the indicated antibodies.

As USP13 is a classic deubiquitinating enzyme, we try to find its substrate by mass spectrometry (MS). We performed silver staining after immunoaffinity purification by USP13 antibody and then used the MS instrument to identify interacting proteins of USP13 (Fig. 3A). MS analysis showed that among the TOP7 interacted proteins (identified total peptides and unique peptides ≥ 2) (Fig. 3B). We selected PCMT1 for further analysis for the following reasons. Firstly, the MS results (Fig. 3C) showed that USP13 interacted with PCMT1. Second, PCMT1 was reported to be overexpressed in PCa tissues [21]. Third, our immunofluorescence staining revealed that endogenous USP13 and PCMT1 have co-localized in the cytoplasm in both PCa cells (Figs. 3D and S3). Moreover, we further found that Flag-tagged PCMT1 and Myc-tagged USP13 could interact with each other in 293 T cells (Fig. 3E) and endogenous USP13 and PCMT1 co-precipitated in both PCa cells (Fig. 3F). Based on binding models of USP13 with PCMT1 (Fig. 3G), the amino residues (Asp78/ His112 were located in the UBP-type zinc finger domain of USP13) of USP3 was forming hydrogen bonds with the matched Arg134/Met98 residues of PCMT1. The USP13 protein was reported to have four domains: ZNF-UBP domain (1–300), USP domain (300–625), UBA1 domain (652–693), and UBA2 domain (727–767) [24, 25]. To map the interacting regions on USP13 and PCMT1, various Myc-tagged USP13 and Flag-PCMT1 deletions were tested (Fig. 3I). IP assays demonstrated that the ZNF-UBP domain (amino acids 1–300) of USP13 interacted with PCMT1, which is consistent with the binding models. Notably, we found that USP13 regulated PCMT1 ubiquitin by K63-linked deubiquitin (Fig. S3B).

### USP13 promotes PCMT1 stability by deubiquitination and positively interacts with PCMT1 in PCa tissues

**Fig. 4:**
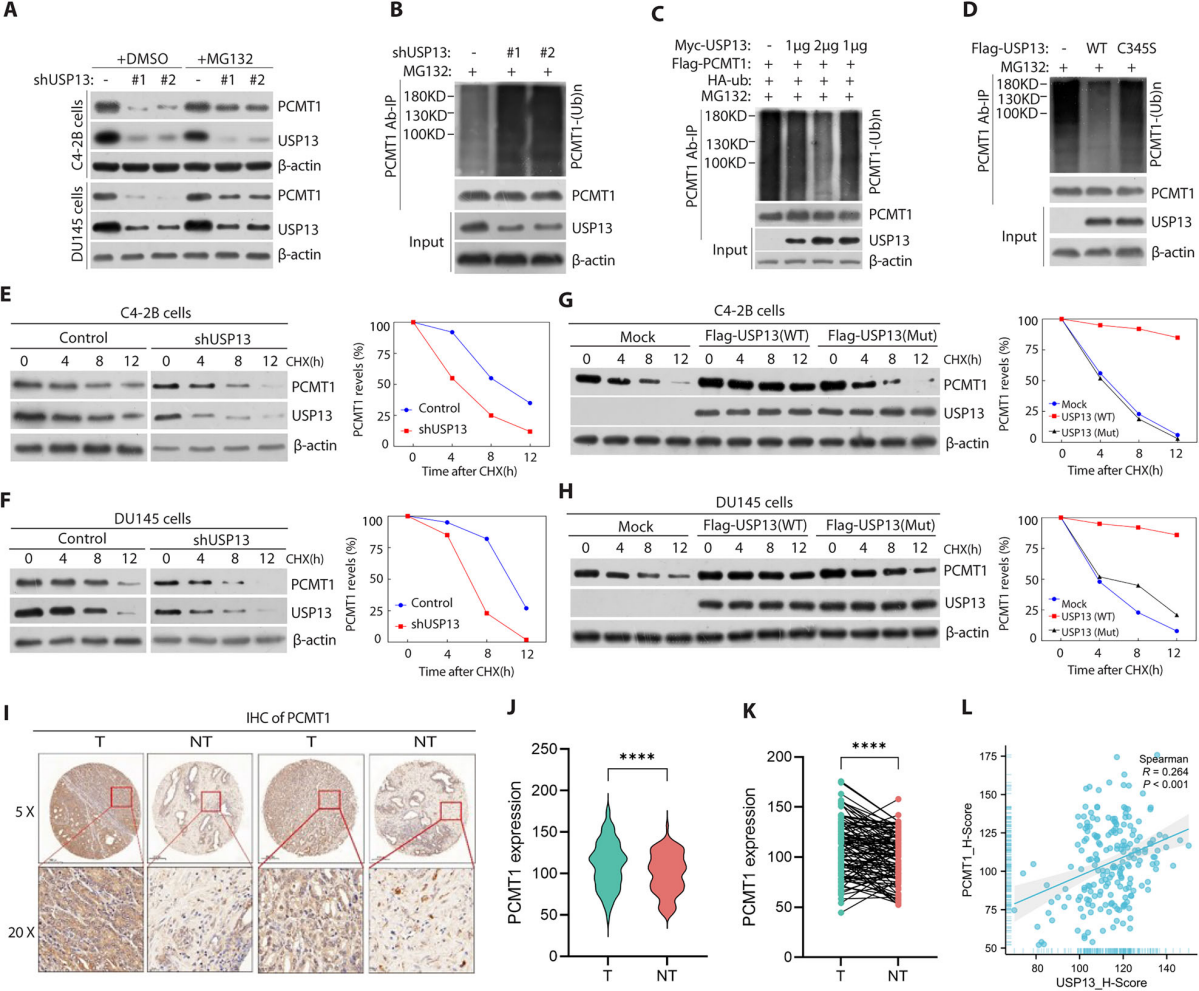
USP13 stabilizes PCMT1 by deubiquitination and is positively related to PCMT1 expression in PCa tissues. **A** Both USP13-silenced C4-2B and DU145 cells were treated with or without MG132, and PCMT1 protein levels were detected by Western Blot. **B** The C4-2B cells stably expressing control or USP13 shRNAs (#1 and #2) were subjected to ubiquitin assay, and the polyubiquitylated PCMT1 protein was detected by the anti-ubiquitin antibody. **C** The HEK293T cells were co-transfected with HA-ubiquitin, Flag-PCMT1, and without or with different dose Myc-USP13 plasmids as indicated. The polyubiquitylated PCMT1 protein was detected by the anti-ubiquitin antibody. **D** The HEK293T cells were transfected with wild-type USP13 plasmids and/or USP13 mutant (C3345S) plasmids for 48h and then treated with MG132 for 6h. The polyubiquitylated PCMT1 protein was detected by the anti-ubiquitin antibody. **E**, **F** The C4-2B cells or DU145 cells were transduced with USP13 shRNAs, treated with 50 mg/mL cycloheximide, harvested at different time points, and then immunoblotted with antibodies to USP13, PCMT1, and β-actin. Right, quantification of PCMT1 protein levels (normalized to β-actin). **G**, **H** The C4-2B cells or DU145 cells were transfected with wild-type Flag-USP7 and or mutation (Flag-USP13 (C345A)) for 48 h, then analyzed and quantified as described in (**E**). **I** Representative staining of PCMT1 in paired adjacent non-tumor tissues (*n* = 99) and matched PCa tissues. **J**, **K** The protein expression of PCMT1 was up-regulated in all PCa tissues (*n* = 99) compared with all adjacent non-tumor tissues (*n* = 99) and or matched adjacent non-tumor tissues. Paired *t*-test, ^****^p < 0.0001. **L** PCMT1 and USP13 protein expression status in adjacent non-tumoree prostate tissue and prostate carcinoma specimens, and the correlation study of PCMT1 and USP13 expression level in PCa tissues. Statistical analyses were undertaken with the χ^2^ test, *p* < 0.001. R, Pearson’s correlation coefficient.

As USP13 is a deubiquitinase ligase, we ask whether and how USP13 regulates PCMT1 stability. Firstly, Western Blot was performed to analyze PCMT1 expression in mice tumor after knocking down of USP13, and we found silencing of USP13 decreased PCMT1 expression in vivo (Fig. S4A). Then, we treated the indicated PCa cells with or without proteasome inhibitor MG132 and detected PCMT1 protein levels (Fig. 4A). We revealed that the treating cells with the proteasome inhibitor MG132 significantly increased the decreased PCMT1 protein level in cells knocking down of USP13 (Fig. 4A). Second, we found that knocking down of USP13 increased PCMT1 polyubiquitination (Fig. 4B) and ectopic expression of USP13 reduced the polyubiquitination of PCMT1 (Fig. 4C), but not the C345S mutant (the catalytically inactive form) (Fig. 4D), suggesting that the enzymatic activity of USP13 is indispensable for USP13-dependent deubiquitination of PCMT1. Then, we test whether USP13 regulates PCMT1 stability. We treated cells with cycloheximide (CHX) and determined the half-life of PCMT1. Figure 4E, F showed that silencing of USP13 decreased the stability of PCMT1 in both PCa cells. Conversely, ectopic expression of wild-type USP13, but not the C345S mutant, increases the stability of endogenous PCMT1 protein, whereas the stability of USP13 was not affected (Fig. 4G, H). We then asked whether PCMT1 was up-regulated in PCa tissue samples. We firstly evaluated the PCMT1 expression in PCa tissue using PCMT1 IHC staining and validated the PCMT1 antibody by showing that it strongly stained PCa tissue sections in contrast to negative staining of adjacent tissue sections by control IgG (Fig. S4B). Imunohistochemical (IHC) was also used to analyze PCMT1 expression in tissue microarray, which includes 100 samples of PCa tissues and 99 samples of adjacent non-tumor tissues (Fig. S4C). The IHC results (Fig. 4I–K) revealed that PCMT1 was also up-regulated in PCa tissues compared with non-tumor tissues, and among the 99 PCa tissue samples, 69 cases had a strong PCMT1 expression while only 30 cases had a weak or negative PCMT1 expression (Table S3). Overexpression of PCMT1 was positively correlated with larger tumor size and poor histological grade, but not correlated with age and Lymph node metastasis (Table S3). Notably, overexpression of USP3 was positively correlated with PCMT1 expression (Fig. 4L). Taken together, upregulation of USP13 promotes PCMT1 stability by deubiquitination and positively correlates with PCMT1 overexpression in PCa tissues.

### USP13 promotes prostate tumor cell proliferation through PCMT1

**Fig. 5:**
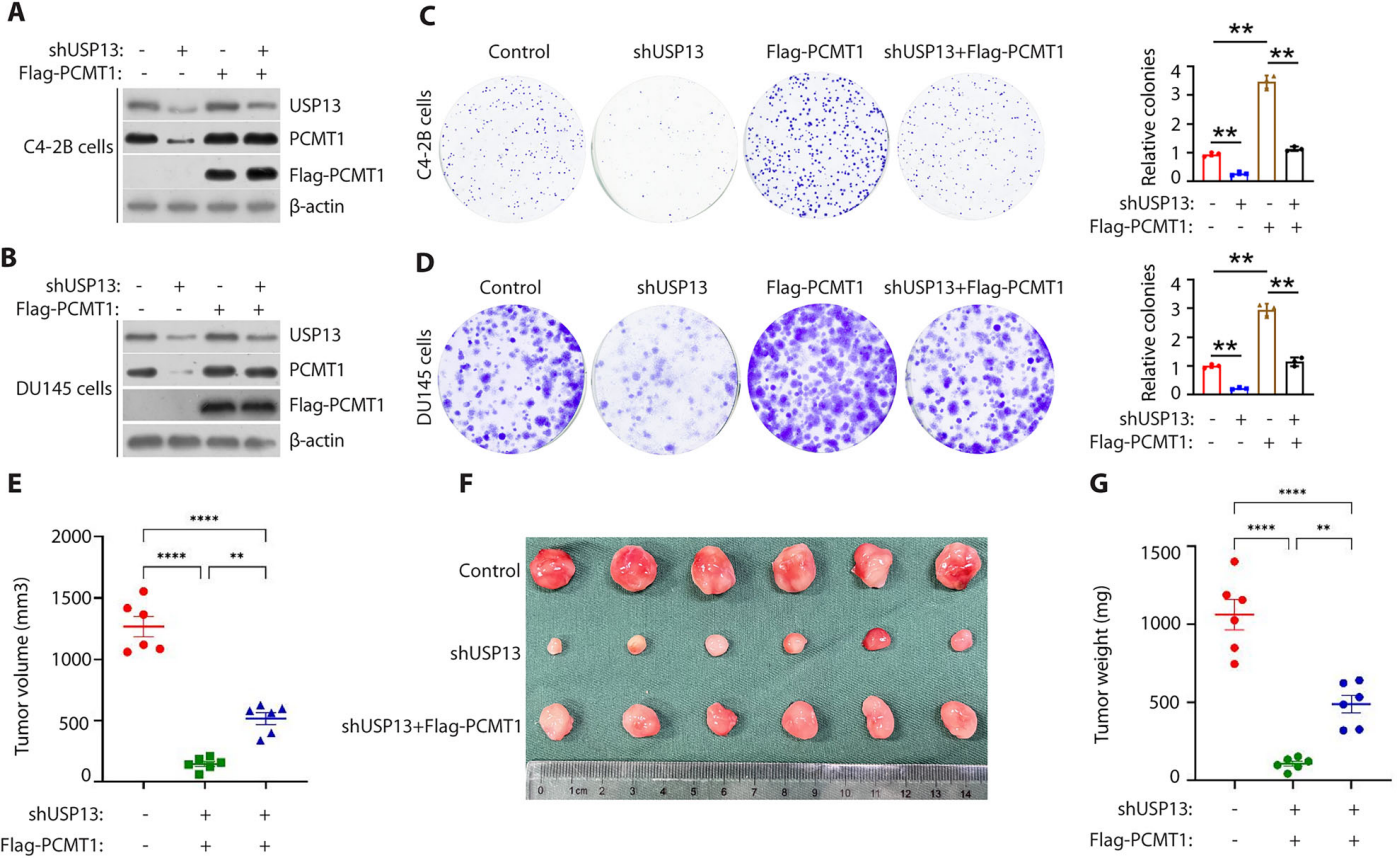
USP13 promotes PCa cell proliferation through PCMT1. **A**, **B** The C4-2B cells or DU145 cells were transfected with the indicated plasmid (Control, shUSP13, Flag-PCMT1, or shUSP7 + Flag-PCMT1), and the corresponding proteins were detected by Western Blot. **C**, **D** The indicated C4-2B cells or DU145 cells were generated as in (**A**), and colony-formation assays were performed. Non-paired *t*-test, ^**^p < 0.01. **E**–**G** The indicated control C4-2B cells or USP13 knocked down C4-2B cells, and or overexpression of PCMT1 on the USP13-silenced C4-2B cells were subcutaneously injected into Balb/c nude mice. Tumor volume (**G**) was measured at 28 days (*n* = 5 per group). After 28 days, mice were sacrificed, and the tumor image (**H**) was presented, and tumor weights (**I**) were measured in the three groups. Statistical significance was determined using one-way ANOVA (^**^p < 0.01 and ^****^p < 0.0001).

To explore the physiologic function of the USP13-PCMT1 axis in cell proliferation, we overexpressed PCMT1 in USP13-silenced PCa cells and detected it by Western Blot. As Fig. 5A, B showed, overexpression of PCMT1 could rescue its protein expression in the USP13-silenced PCa cells. Then, we performed a colony formation assay in these cells and found that PCMT1 overexpression promoted colony formation and significantly rescued the effect of USP13 knockdown in two PCa cells (Fig. 5C, D). To test whether the USP13-PCMT1 axis modulates AR transcriptional activity, we knocked down USP13 and overexpressed PCMT1 in USP13-silenced Rv1 cells, and then detected AR target genes. We found that silencing of USP13 reduced AR target genes (PSA, NKX3.1, and KLK2) and overexpression of PCMT1 could rescue this effect (Fig. S5A), suggesting that USP13 may modulate AR transcriptional activity through regulating PCMT1. PCMT1 has been reported to promote prostate cancer progression via the PI3K/AKT/GSK-3β pathway [21]. We test whether the USP13-PCMT1 axis regulates the PI3K/AKT/GSK-3β pathway. We found silencing of USP13 or PCMT1 reduced the phosphorylation of AKT, but did not change the total AKT and GSK-3β expression, and overexpression of PCMT1 can rescue this effect (Fig. S5B). These suggest USP13 regulates the PI3K/AKT pathway through PCMT1. To test the biological function of the USP3-PCMT1 axis in vivo, we employed an orthotopic prostate tumor model in which C4-2B cells were injected subcutaneously into the nude mice. Silencing of USP13 dramatically decreased tumor growth, and co-overexpression of PCMT1 significantly rescued this effect (Fig. 4E–G). Collectively, these data suggest that USP13 regulates prostate tumor cell proliferation in a PCMT1-dependent manner.

### Targeting USP13 inhibits enzalutamide-resistant prostate cancer growth in vitro and in vivo

**Fig. 6:**
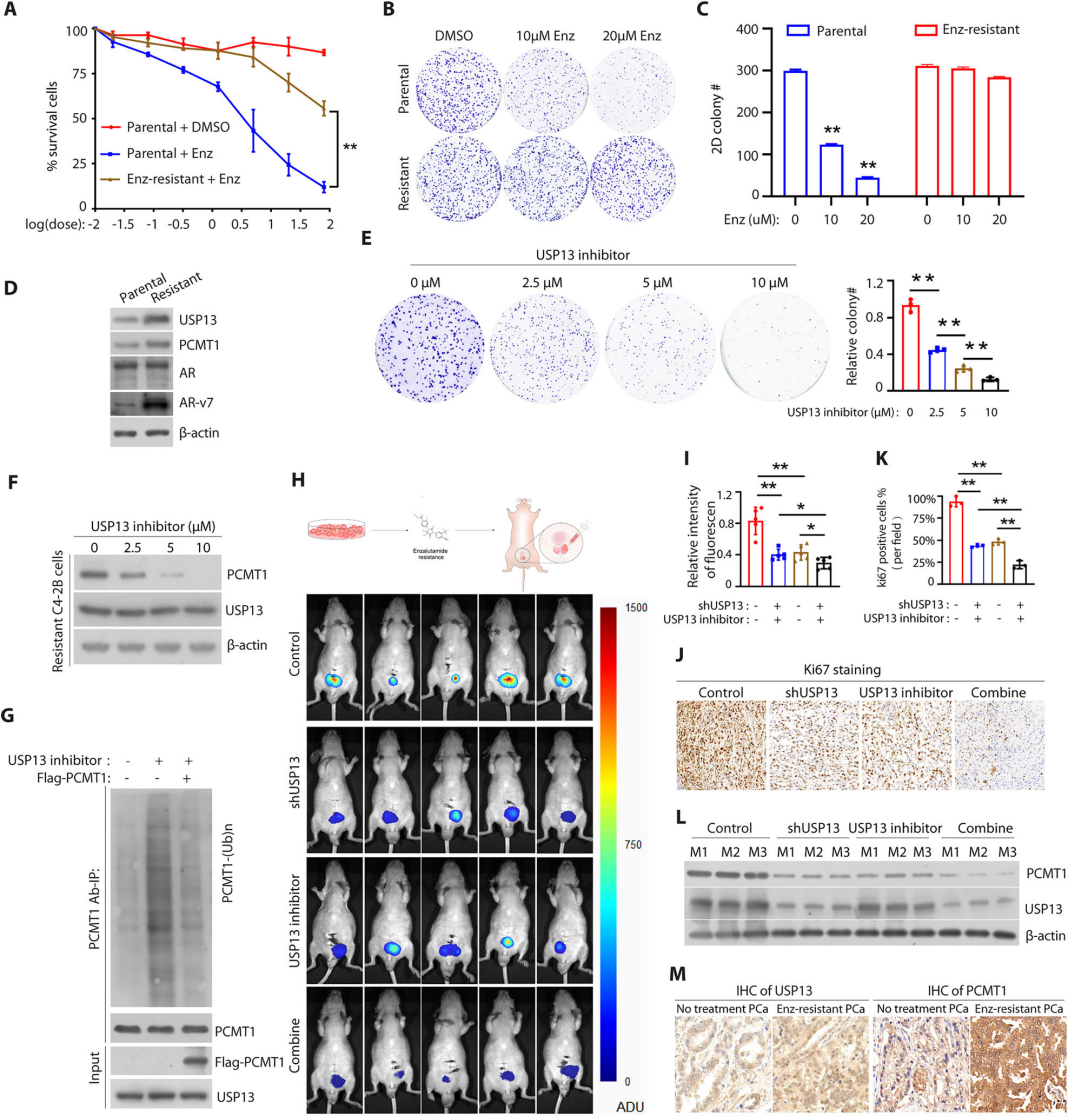
Targeting USP13 promotes PCa cells sensitive to enzalutamide. **A** Survival of enzalutamide-resistant or parental C4-2B cells after treatment with increasing amounts of enzalutamide for 72 h. *Y* axis is the % of surviving cells as measured by the MTT assay, and *X* axis is the log-transformed dosage of enzalutamide (μM). **B**, **C** Colony formation of enzalutamide-resistant or parental C4-2B cells in the presence of enzalutamide. Cells were seeded on the cell-culture plate at low density, and then treated with the indicated concentration of enzalutamide. The number of colonies was scored after 2 weeks. Non-paired *t*-test, ^**^p < 0.01. **D** Western Blot was used to detect the indicated proteins in the enzalutamide-resistant or parental C4-2B cells. **E**, **F** The enzalutamide-resistant C4-2B cells were seeded on the cell-culture plate at low density, and then treated with a dose of USP13 inhibitors for 7 days, and then analyzed by western blotting for the indicated proteins. Non paired it-test, ^**^p < 0.01. **G** The control enzalutamide-resistant C4-2B cells, enzalutamide-resistant C4-2B cells treated with USP13 inhibitors, and or overexpression of PCMT1 on USP13 inhibitors-treated enzalutamide-resistant C4-2B cells were subjected to ubiquitin assay, and the polyubiquitylated PCMT1 protein was detected by the anti-ubiquitin antibody. **F**–**I** The mice bearing enzalutamide-resistant C4-2B tumors and the USP13-silenced mice bearing enzalutamide-resistant C4-2B tumors were treated with vehicle control and USP13 inhibitor (10 mg/kg i.p.) two times per 5 days for 25 days. On 25 days, mice were injected with luciferin, and tumor formation was monitored using in vivo bioluminescence imaging. Statistical significance was determined using one-way ANOVA (^*^p < 0.05 and ^**^p < 0.01). **J**, **K** Example staining of Ki67 on the tumor sections derived from the above treatment mice. The staining was developed by DAB (brown) and counterstained by hematoxylin (blue). The percentage of Ki67-positive cells in the staining was analyzed. Non-paired *t*-test, ^*^p < 0.05 and ^**^p < 0.01. **L** Western Blot was performed to analyze PCMT1, USP13, and β-actin protein expression in the above mice tumor (*n* = 3). **M** lHC staining of USP13 and PCMT1 on the non-enzalutamide treatment PCa tissue and enzalutamide-resistant PCa tissues.

Although enzalutamide (ENZ) is effective at first, resistance to next-generation anti-androgen enzalutamide constitutes a major challenge for the treatment of castration-resistant prostate cancer (CRPC). In this study, we used a widely approach [26–29] that we generated enzalutamide-resistant C4-2B cells by growing parental C4-2B cells with gradually increasing concentrations of enzalutamide (1–20 uM) for 3 months and we confirmed that enzalutamide treatment indeed had much less effect on proliferation of resistant C4-2B cells than parental cells in both the MTT (Fig. 6A) and colony formation assays (Fig. 6B, C). In the enzalutamide-resistant C4-2B cell line, we found increased levels of UISP13, PCMT1, and AR splicing variant AR-v7, but not AR, relative to parental cells (Fig. 6D). To globally characterize the downstream regulatory network governed by the USP13-PCMT1 axis in enzalutamide-resistant prostate cancer cells, we performed RNA-sequencing (RNA-seq) in enzalutamide-resistant C4-2B with stable knockdown of USP13 (shUSP13), PCMT1 (shPCMT1), and non-targeting control (NC). As shown in Fig. S6A and Table S4, Venn diagram analysis identified overlapping differentially down-regulated 71 expressed genes (DEGs) between shUSP13 vs NC and shPCMT1 vs NC, which represent the core downstream targets of the USP13–PCMT1 axis. KEGG pathway enrichment analysis of these overlapping DEGs (Fig. S6B) revealed significant enrichment of pathways related to drug resistance, metabolism, and inflammation, including the HIF-1 signaling pathway, cAMP signaling pathway, endocrine resistance, and purine metabolism. Gene set enrichment analysis (GSEA) further confirmed that knockdown of USP13 and PCMT1 significantly suppressed multiple critical biological programs (Fig. S6C–S6G), including: HIF-1 signaling pathway (inflammatory and stress-related signaling) (Fig. S6C), cAMP signaling pathway (resistance-related pathways) (Fig. S6D), endocrine resistance (Fig. S6E), alanine metabolism (Fig. S6F), and purine metabolism (metabolic enzyme pathways) (Fig. S6G). These results clearly demonstrate that the USP13-PCMT1 axis globally orchestrates a broad spectrum of malignant programs encompassing drug resistance, metabolic reprogramming, and inflammatory signaling in enzalutamide-resistant prostate cancer.

**Fig. 7:**
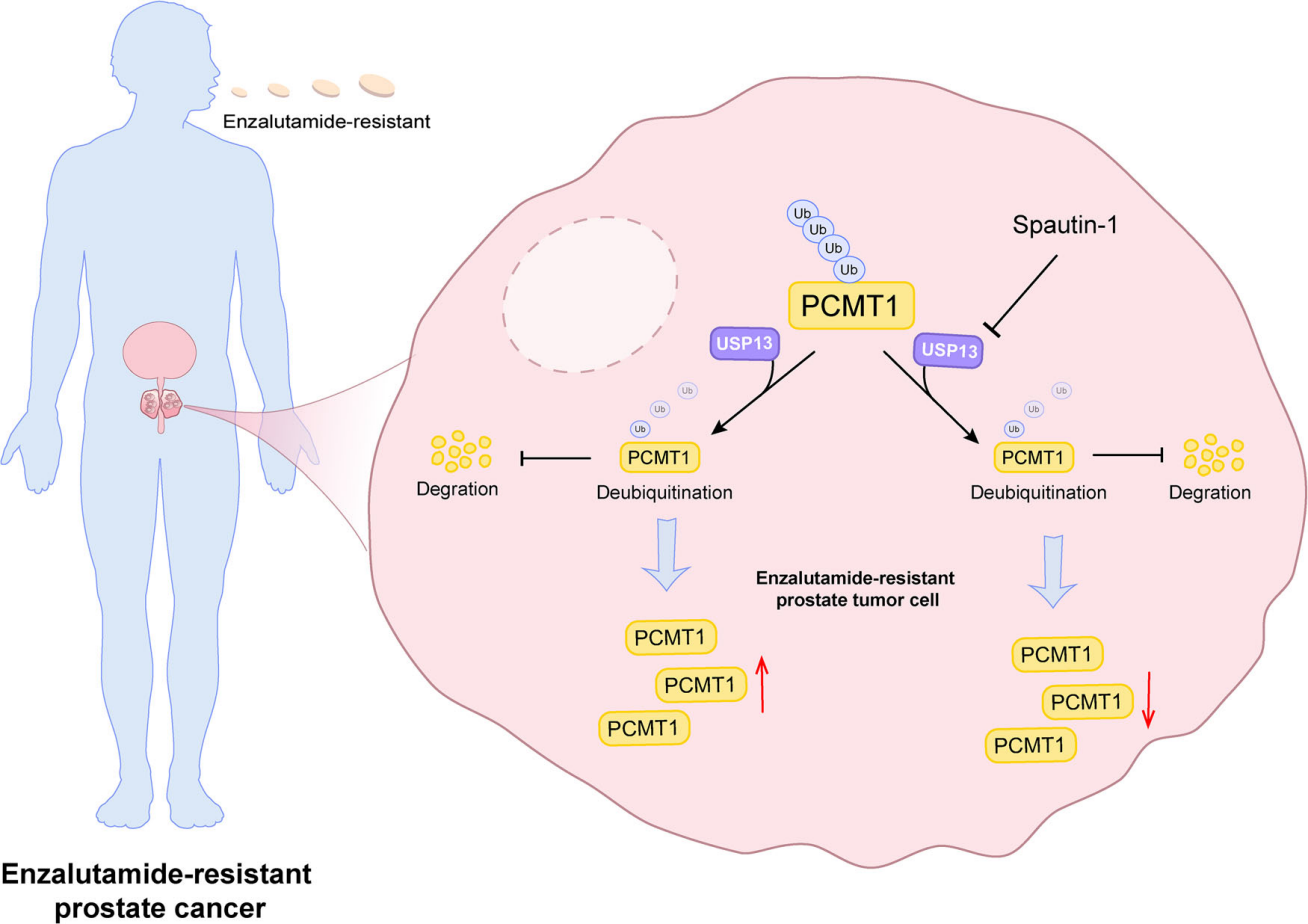
Targeting USP13 by Spautin-1 reduces enzalutamide-resistant tumor growth. Proposed pathway of USP13-PCMT1 in next-generation anti-androgen resistance and prostate cancer progression. Up-regulation of USP13 stabilizes PCMT1 by deubiquitination. USP13 inhibition by Spautin-1 inhibits enzalutamide-resistant tumor growth.

Spautin-1 is a pharmacological inhibitor of USP13 that functions through binding to the USP13 active site and suppressing its deubiquitinating enzyme activity [8, 30–32]. Then, we test whether USP13 inhibitors inhibit enzalutamide-resistant C4-2B cells. We observed Spautin-1 treatment significantly suppressed the growth of the enzalutamide-resistant C4-2B cells (Fig. 6E) and also decreased PCMT1 expression (Fig. 6F) in a dose-dependent manner. Moreover, inhibition of USP13 by Spautin-1 decreased PCMT1 polyubiquitination, and ectopic expression of PCMT1 significantly rescued this effect (Fig. 6G). To examine if inhibition of USP13 inhibits enzalutamide-resistant prostate cancer growth in vivo, we generated an enzalutamide-resistant tumor derived from enzalutamide-resistant C4-2B cells (Fig. 6H). We demonstrated that silencing of USP13 or pharmacological inhibition of USP13 significantly reduced enzalutamide-resistant tumor growth, while combination treatment further inhibited tumor growth (Fig. 6H and I). Immunohistochemical staining of Ki67 showed that cell proliferation was significantly inhibited by silencing of USP13 or pharmacological inhibitor of USP13 alone and further inhibited by the combination treatments (Fig. 6J, K). Additionally, the expression of PCMT1 and USP13 was further confirmed in the enzalutamide-resistant tumor model (Fig. 6L). Notably, we found that USP13 and PCMT1 proteins were higher expressed in the enzalutamide-resistant PCa tissue compared with treatment-naïve PCa tissue (Fig. 6M and S6H, S6I). What is the upstream driver of USP13? It has been reported that Hypoxia induced USP13 expression [33]. To further investigate this, we firstly used hypoxic conditions and or overexpression of HIF-1α to treat the enzalutamide-resistant C4-2B cells. We found that hypoxic conditions and or overexpression of HIF-1α can induce the upregulation of both USP13 and PCMT1 (Fig. S6J). To further confirm this, we constructed luciferase reporter plasmids containing the USP13 promoter. Luciferase assays revealed that silencing of HIF-1α decreased USP13 transcriptional activity (Fig. S6K). These results suggest that Hypoxia induces USP13 overexpression. Furthermore, we established patient-derived xenograft (PDX) models, and we found that USP13 knockdown significantly reduced tumor size (Fig. S6L). USP13 has been reported to promote the degradation of the inflammation molecule STING and prevent the recruitment of TBK1 to the signal complex [34, 35]. So, we detected the inflammation molecules STING and TBK1 from tumors, as shown in Fig. S6L by Western Blotting. We found that USP13 knockdown increased the inflammation molecule STING protein from the PCa patient-derived xenograft (PDX) models (Fig. S6L) and overexpression of PCMT1 can rescue this effect (Fig. S6M), but not change the inflammation molecule TBK1 protein. Taken together, these results suggest that targeting USP13 by Spautin-1 reduces enzalutamide-resistant tumor growth (Fig. 7).

## Discussion

Drug resistance to molecularly targeted therapies is a current challenge in cancer treatments. Recent years have seen an explosion of research on the role of the ubiquitin (Ub) proteasome system (UPS), which is involved in a cohort of cellular processes, such as cell division, gene expression, and DNA repair, through maintaining the equilibrium between ubiquitination and deubiquitination [36, 37]. Targeting DUB may be a promising approach to inhibit drug-resistant cancers. Here, we found that the deubiquitinating enzyme USP13 was up-regulated in PCa, and overexpression of USP13 was positively correlated with larger tumor size and poor histological grade. USP13 knockdown significantly reduced PCa cell proliferation in vitro and vivo. These support that USP13 is a tumor promoter in PCa. We further showed that USP13 was increased in enzalutamide-resistant PCa cells, implying that USP13 is involved in drug resistance in cancer.

The human PCMT1 is a protein methyltransferase that exists widely in the human body, which contains an RNA-binding domain, a methyltransferase domain, and a *S*-adenosylme-thionine (SAM) binding domain [38, 39]. PCMT1 catalyzes the transfer of methyl groups of SAM to the side chain carboxyl groups of L-isoaspartic acid and D-aspartic acid residues [40]. Recent studies reported that PCMT1 was overexpressed in PCa, and overexpression of PCMT1 may be an important factor that allows tumor cells to survive longer than normal cells [23]. However, it is still unclear which mechanism leads to PCMT1 overexpression. Here, we identify that the ZNF-UBP domain of USP13 interacts with PCMT1 and promotes its stability by deubiquitinating. In clinic PCa tissues, we demonstrated that high expression of USP13 was positive with the PCMT1 overexpression. Notably, we found that USP13 contributes to PCMT1 overexpression in enzalutamide-resistant PCa cells. Our results suggest that abnormal overexpression of PCMT1 may lead to the occurrence of EnzR CRPC. Suppressing PCMT1 expression may delay PCa progression. In addition, the result reflected that PCMT1 inhibitors could be potential drugs to treat EnzR CRPC in the future.

Enzalutamide, a second-generation AR inhibitor, is indicated for the treatment of castration-resistant prostate cancer (CRPC) in numerous countries worldwide [27, 41]. However, by constitutively sustaining AR signaling, expression of ligand-binding domain truncated AR variants accounts for the major mechanism underlying the resistance to anti-androgen drugs [42–44]. A study reported that USP13 is overexpressed in PCa tumors and a significant correlation between the expression of USP13 and the AR pathway [45], suggesting that USP13 may regulate the AR pathway. Small-molecule inhibitor spautin-1, a pharmacological inhibitor of USP13, binds to the USP13 active site and suppresses its deubiquitinating enzyme activity [31, 46, 47]. However, it is still unclear whether it could be used to target enzalutamide-resistant prostate cancer. Here, we provide a proof of concept by using USP13 inhibitor (spautin-1) to target enzalutamide-resistant prostate cancer. We observed inhibition of the USP13-PCMT1 signaling pathway in resistant cells, which re-sensitized the resistant cells to enzalutamide treatment, and spautin-1 could decrease PCMT1 expression by inhibiting the enzymatic activity of USP13. Notably, tumors that are resistant to enzalutamide are highly sensitive to spautin-1 treatment. spautin-1 significantly enhanced enzalutamide treatment both in vitro and in vivo. Our promising preclinical data shed light on future clinical trial development by using the USP13 inhibitor in combination with enzalutamide in advanced prostate cancer treatment.

Current therapeutic strategies are aimed at targeting AR-FL but are unlikely to have significant effects in prostate cancers that express AR splicing variants in enzalutamide-resistant prostate cancer. Our findings showed that upregulation of USP13 stabilizes PCMT1 by deubiquitination, which promotes enzalutamide-resistant PCa, and inhibition of USP13 by Spautin-1 significantly decreases enzalutamide-resistant tumor growth, which suggests that targeting USP13 may be a promising strategy to target advanced stages of prostate cancer.

## Supplementary information

Supplementary data

original Western blots

## Data Availability

The datasets used and/or analyzed during the current study are available from the corresponding author on reasonable request.
