# Supplementary material for: USP13 promotes enzalutamide resistance by catalyzing depolyubiquitination of PCMT1 in prostate cancer
Source: Cell Death Dis. 2026 Apr 30;17(1):576. doi: 10.1038/s41419-026-08824-9 (PMC13272797; doi:10.1038/s41419-026-08824-9)
Supplement: Supplementary file 1 — Supplementary data [file 41419_2026_8824_MOESM1_ESM.doc]

**Supplementary Data**

**Supplemental Figures, Tables, Materials and Methods, References**

**Supplementary Figures**

**Fig. S1**

**
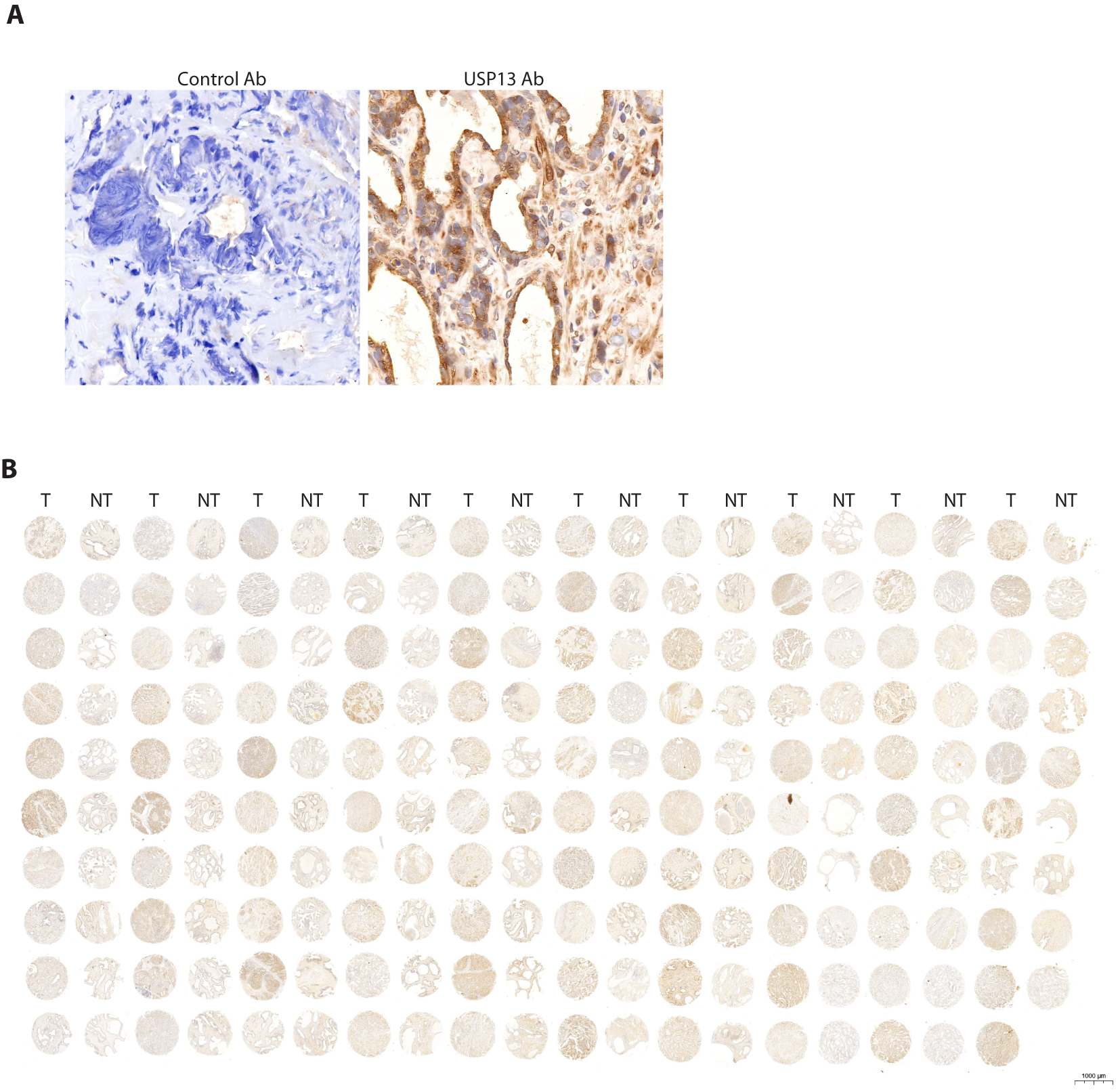
**

**Fig. S1**

(A) IHC staining of USP13 on the adjacent PCa tissue sections using control IgG (left) and USP13 antibody (right). (B) IHC staining of a PCa tissue microarray including 199 cases with an anti-USP13 antibody. NT: adjacent normal tissue, T: tumor tissue.

**Fig. S2**

**
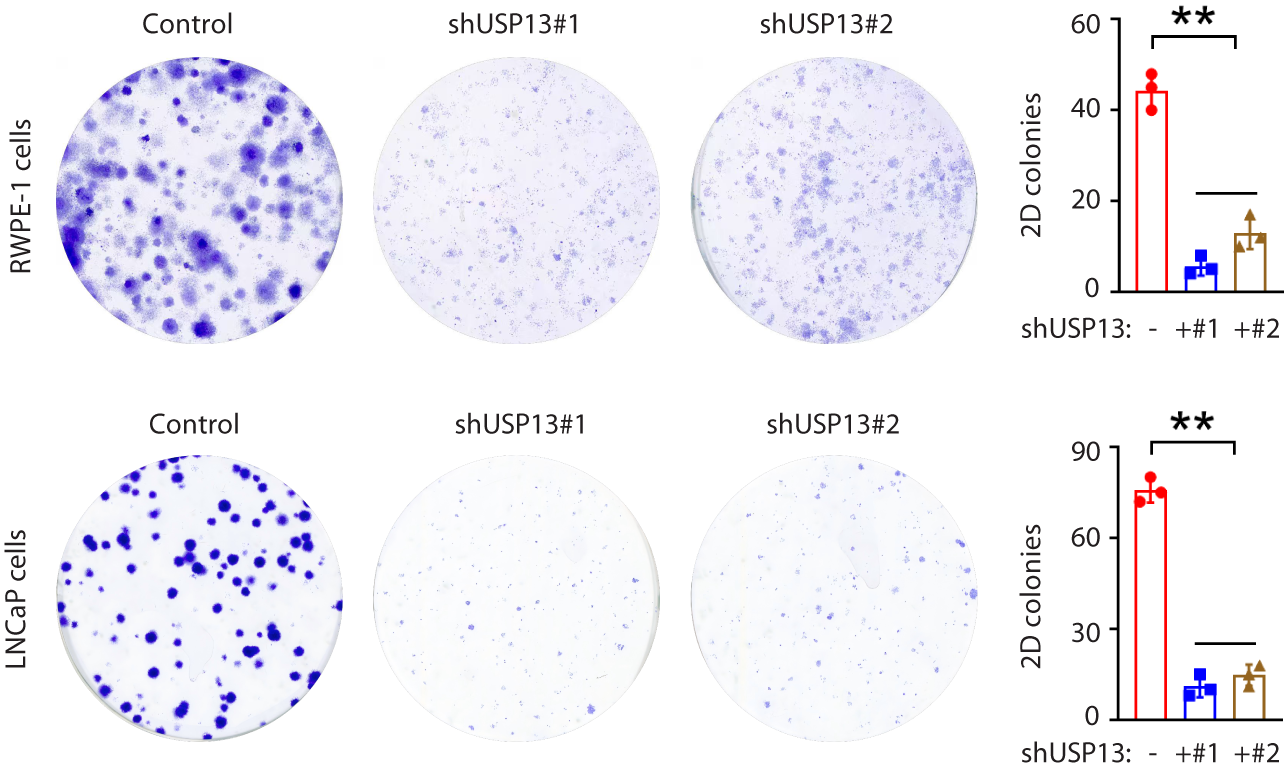
**

**Fig. S2**

The effects of the stable silencing of USP13 on RWPE-1 and LNCaP cell colony formation were determined.

**Fig. S3**

**
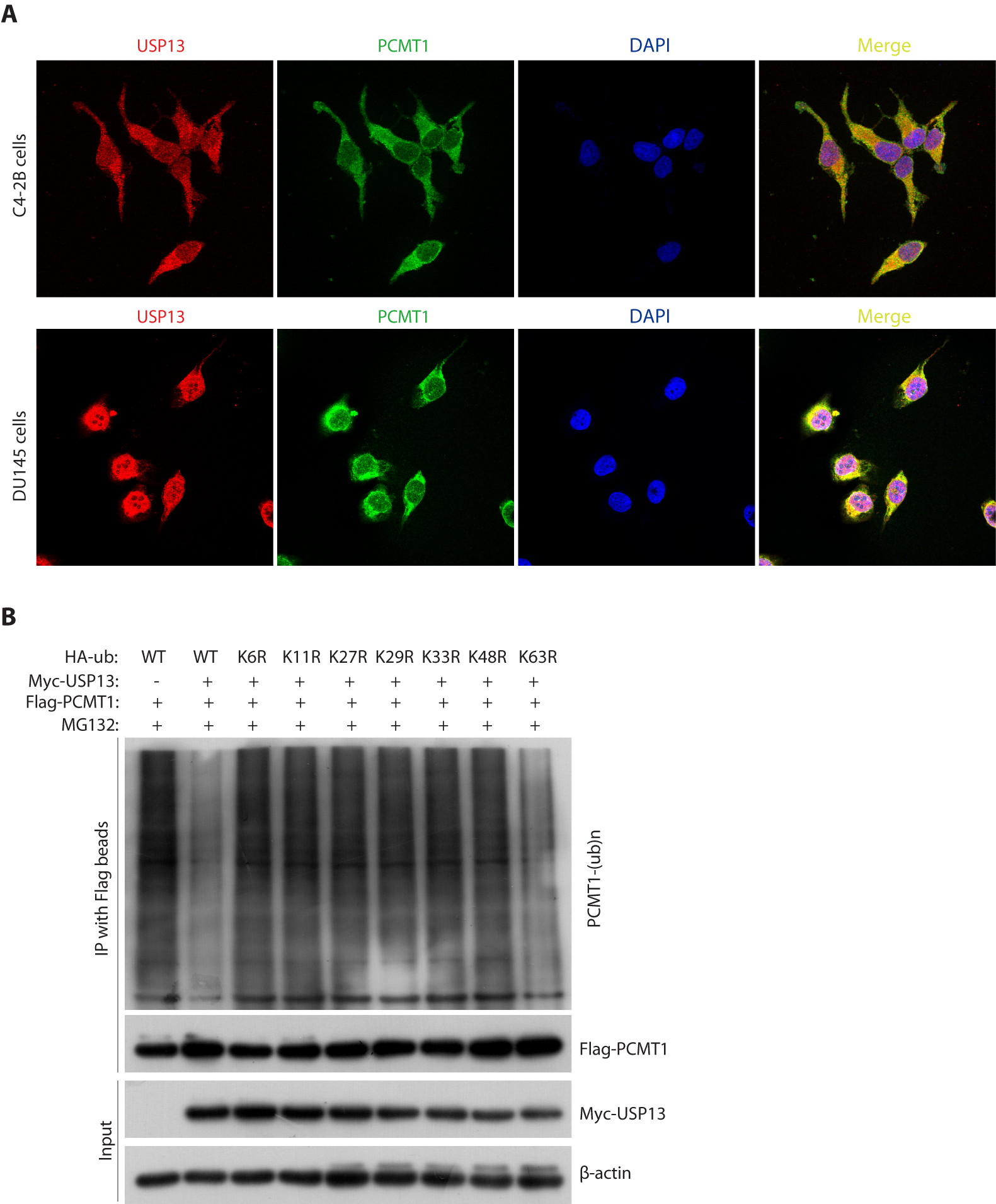
**

**Fig. S3**

(A) Fluorescent images of PCMT1 (green) and USP13 (red) were immunostained using their antibody in C4-2B and DU145 cells. Nuclear 4', 6-diamidino-2-phenylindole (DAPI; blue) was used to stain nucleus. (B) HEK293T cells were transfected with Flag-PCMT1, Myc-USP13, and HA-ub (wild type and or mutant). The polyubiquitylated PCMT1 protein was detected by the anti-Ub antibody.

**Fig. S4**

**
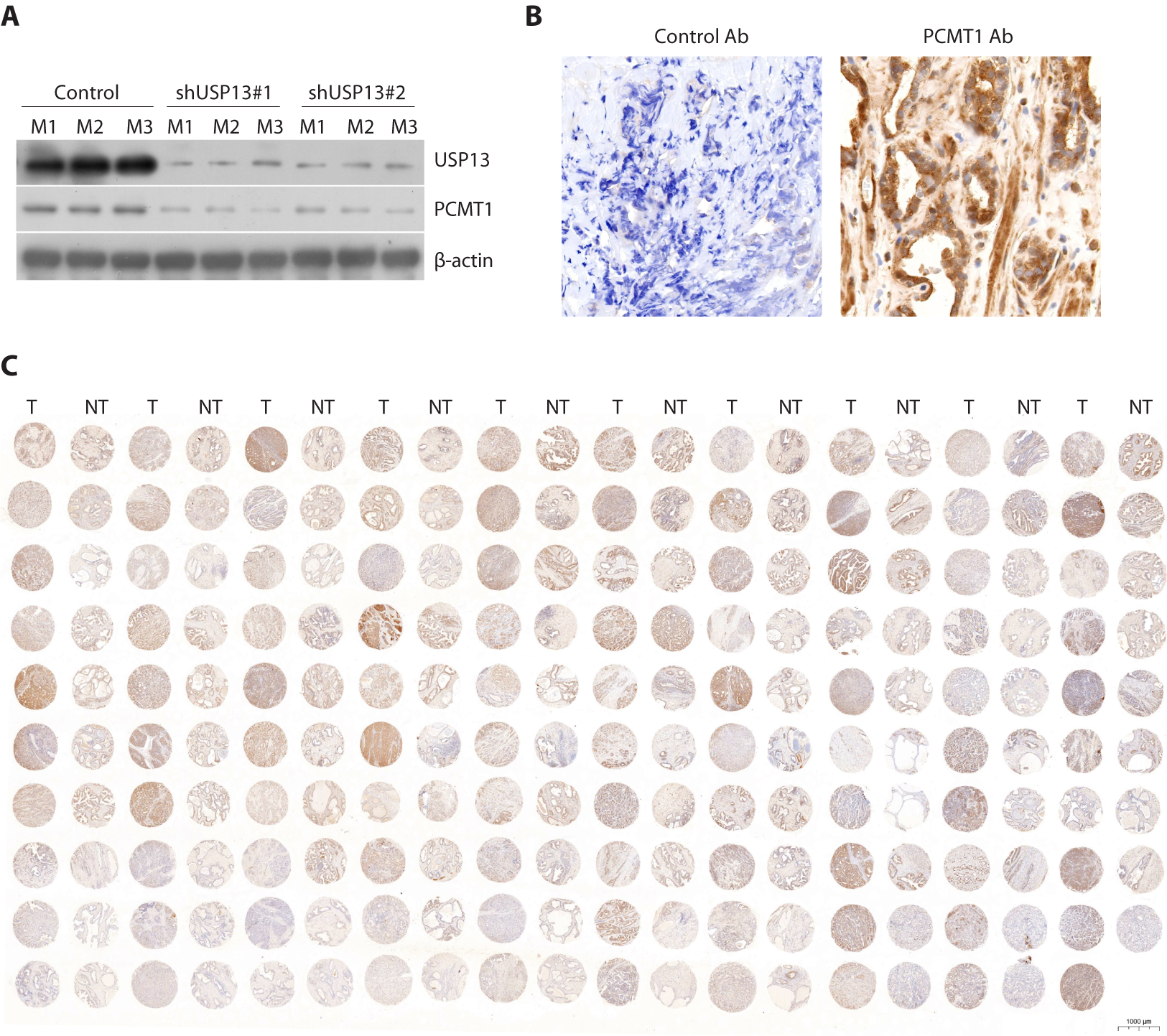
**

**Fig. S4**

(A) Western Blot was performed to analysis of PCMT1, USP13 and β-actin protein expression in mice tumor (n = 3) from Figure 2H. (B) IHC staining of PCMT1 on the adjacent PCa tissue sections using control IgG (left) and PCMT1 antibody (right). (C) IHC staining of a PCa tissue microarray including 199 cases with an anti-PCMT1 antibody. NT: adjacent normal tissue, T: tumor tissue.

**Fig. S5**

**
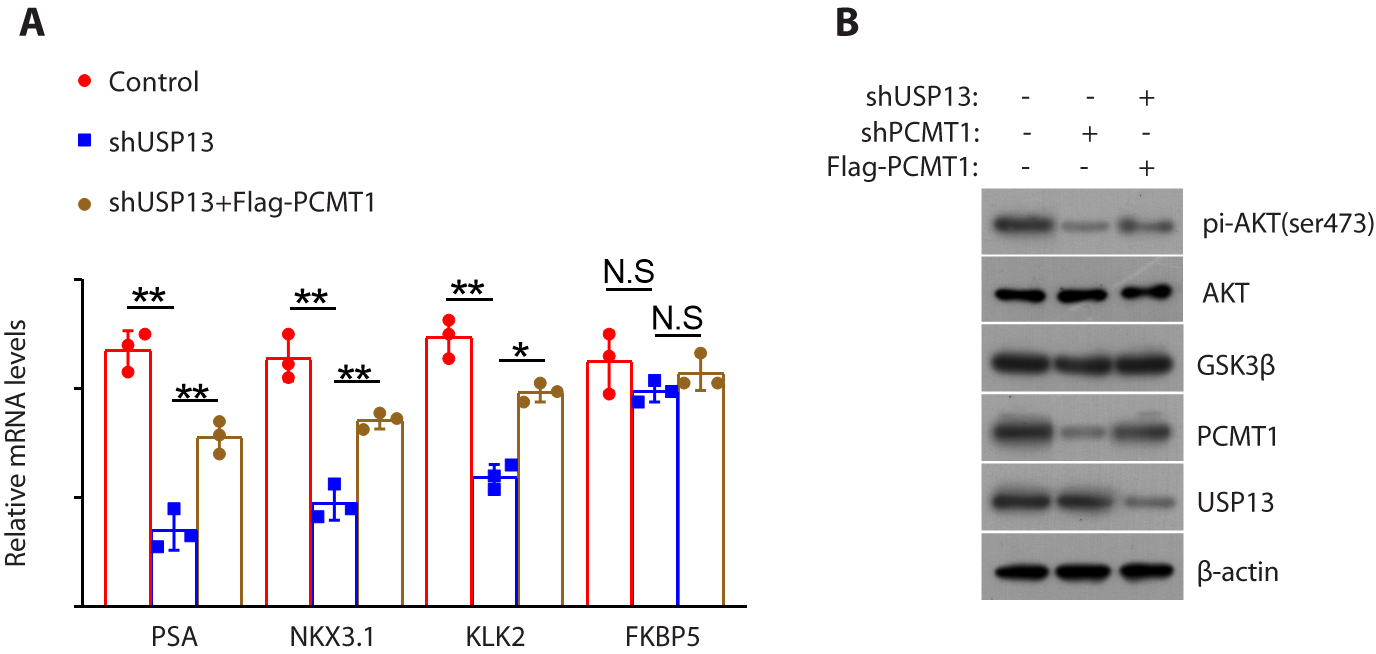
**

**Fig. S5**

(A) Silencing of USP13 and overexpression of PCMT1 in USP13 silenced Rv1 cells, and then detected the indicated selected AR target genes by qRT-PCR. ^*^*p* < 0.05; ^**^*p* < 0.01. (B) Silencing of USP13 or PCMT1 reduced the phosphorylation of AKT (ser473), but not change the total AKT and GSK-3β expression, and overexpression of PCMT1 can rescue this effect.

**Fig. S6**


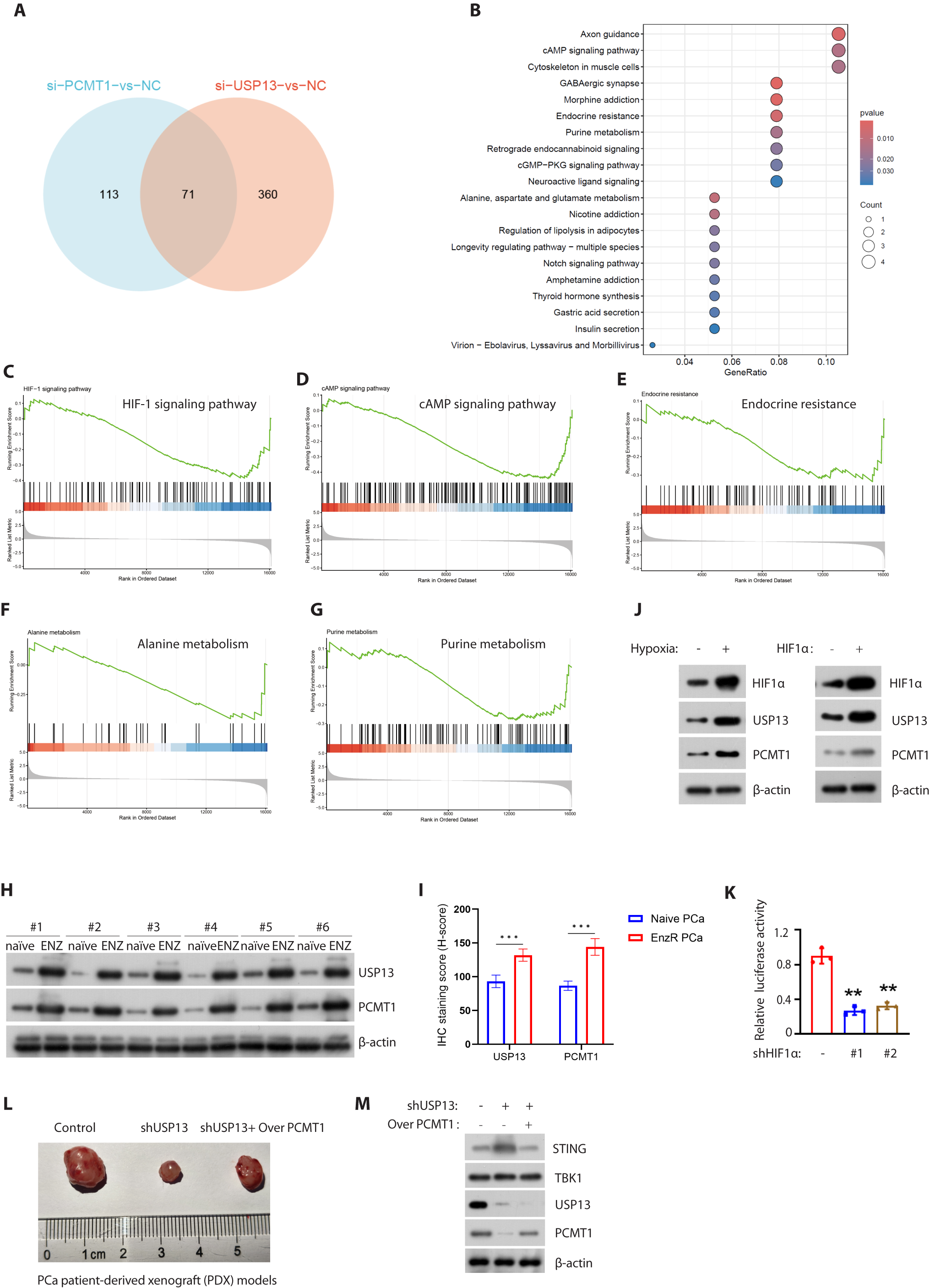


**Fig. S6**

(A) Volcano plot showing the differentially down-regulated expressed 71 genes (DEGs) (fold change ≥ 2) between USP13 and PCMT1 silenced enzalutamide-resistant C4-2B in the RNA-seq analysis. (B) KEGG pathway enrichment analysis of these overlapping DEGs revealed significant enrichment of pathways related to drug resistance, metabolism, and inflammation, including HIF-1 signaling pathway, cAMP signaling pathway, endocrine resistance and purine metabolism. (C-G) Gene set enrichment analysis (GSEA) further confirmed that knockdown of USP13 and PCMT1 significantly suppressed multiple critical biological programs, including: HIF-1 signaling pathway (inflammatory and stress-related signaling), cAMP signaling pathway (resistance-related pathways), Endocrine resistance, Alanine metabolism and purine metabolism (metabolic enzyme pathways). (H) Western blotting was used to detect USP13 and PCMT1 on treatment-naïve PCa tissue and enzalutamide-resistant PCa tissues. (I) lHC staining of USP13 and PCMT1 on on treatment-naïve PCa tissue and enzalutamide-resistant PCa tissues, and the result was performed for statistic. ^*^*p* < 0.05; ^**^*p* < 0.01. (J) Hypoxia or HIF-1α overexpression induces up-regulation of PCMT1 and USP13. (K) Luciferase assays revealed that silencing of HIF-1α promoted USP13 transcriptional activity. (L) (M) Tissue samples from PCa patients were digested into single cells and then the cells were infected virus of only USP13 knockdown and or combined PCMT1 overexpression plasmids. The proteins form the above the tumors were subjected to detect the indicated proteins by Western Blotting.

**Supplementary Tables**

**Supplementary Table 1. Clinic pathological variables, USP13 expression in prostate cancer patients from the Platform.**

Variables All USP13 *P*

n=99 n=31 (low) n=68 (High) value^#^

Age

≤ 65 10 13 0.1511

＞ 65 21 55

Tumor stage

T1-T2 18 25 0.0474^*^

T3-T4 13 43

Stage

I-II 14 14 0.0118^*^

III-IV 17 54

Gleason grade

≤ 7 15 17 0.0210^*^

> 7 16 51

Lymph node metastasis

No 31 65 0.5498

Yes 0 3

^#^*P* value was analyzed by chi-sequared test; ^*^ indicates *P* < 0.05, ^**^*P* < 0.01 with statistical significance.

**Supplementary Table 2.** **The candidate ubiquitin-proteins interacted with USP13 identified by Mass Spectrometry.**

| Protein Names | Gene Names | #Peptides | Unique Peptides | Mol. Weight [kDa] |
| --- | --- | --- | --- | --- |
| Ubiquitin carboxyl-terminal hydrolase 13 | USP13 | 37 | 37 | 97.327 |
| Ubiquitin-60S ribosomal protein L40 | | UBA52 | | --- | | 5 | 5 | 14.728 |
| Protein-L-isoaspartate(D-aspartate) O-methyltransferase | PCMT1 | 3 | 3 | 24.636 |
| Heterogeneous nuclear ribonucleoprotein H | HNRNPH1 | 3 | 3 | 49.229 |
| Scaffold attachment factor B1 | SAFB | 3 | 3 | 102.642 |
| SAFB-like transcription modulator | SLTM | 2 | 2 | 117.149 |
| Protein S100-A9 | S100A9 | 2 | 2 | 13.242 |
| T-complex protein 1 subunit eta | CCT7 | 2 | 2 | 59.367 |

**Supplementary Table 3.** **Clinic pathological variables, PCMT1 expression in prostate cancer patients from the Platform.**

Variables All PCMT1 *P*

n=99 n=30 (low) n=69 (High) value^#^

Age

< 65 11 13 0.0572

≥ 65 19 56

Tumor stage

T1-T2 19 25 0.0126^*^

T3-T4 11 44

Stage

I-II 15 21 0.0629

III-IV 15 48

Gleason grade

≤ 7 11 21 0.0430^*^

> 7 19 48

Lymph node metastasis

No 28 68 0.1640

Yes 2 1

^#^*P* value was analyzed by chi-sequared test; ^*^ indicates *P* < 0.05, ^**^*P* < 0.01 with statistical significance.

**Supplementary Table 4.** **Identified overlapping differentially down-regulated 71 expressed genes between shUSP13 vs NC and shPCMT1 vs NC.**

|  | **si-USP13-vs-NC** | | **si-PCMT1-vs-NC** | |
| --- | --- | --- | --- | --- |
| **gene_id** | **FoldChange** | **p-value** | **FoldChange** | **p-value** |
| ABAT | 0.2324699048385575 | 7.404963555423448e-06 | 0.3338830274550556 | 6.430238379451282e-05 |
| ABCD2 | 0.2490907883860424 | 0.0001230889242566 | 0.4052921082780217 | 0.0069176066866921 |
| ADCY5 | 0.2490839701682353 | 1.688597935231141e-05 | 0.3492659982847964 | 0.0001447572807379 |
| AMPD3 | 0.4462420884722058 | 7.910362329113794e-08 | 0.2755717524398622 | 5.1003532424114486e-14 |
| ARLNC1 | 8.175170174137905 | 0.0034478434440965 | 7.482025647993649 | 0.0060795621492155 |
| ATP1A3 | 0.2013857536744638 | 2.097859301066212e-25 | 0.3812348578829944 | 1.0798565947809742e-11 |
| B3GALT4 | 0.4622154325505966 | 0.0071254642272002 | 0.4514153294877498 | 0.0051583136008879 |
| C11orf86 | 0.3031463078438202 | 0.0012904282820559 | 0.4045401402635476 | 0.0132698835490088 |
| CA12 | 0.2164336430471133 | 2.6626446928049474e-09 | 0.4591371148467594 | 0.0010726558263272 |
| CCDC160 | 0.4525645229198744 | 0.0083214053976448 | 0.4654867734562589 | 0.0094520455145994 |
| CEND1 | 0.0985419708914884 | 4.669758659551242e-09 | 0.2137788990917382 | 4.668299046677149e-06 |
| COL5A2 | 3.5202724633913 | 2.419149367227232e-06 | 2.236791487812796 | 0.0072130057433341 |
| DACT3 | 0.402779594072372 | 0.0154936287279819 | 0.2931498180524283 | 0.0010909792050517 |
| DLL1 | 0.2788826014290456 | 1.3464349687155918e-10 | 0.3438441617030865 | 2.6013603163962777e-07 |
| DTX4 | 0.3386149852248103 | 5.7104475160064506e-08 | 0.4382759058224457 | 1.109687883611011e-05 |
| EGLN3 | 0.2604460481532594 | 0.015491680619008 | 0.1308306782048672 | 0.0019823635030477 |
| EID3 | 0.4906688734917313 | 0.001041099310785 | 0.4874377703276035 | 0.0015677220510605 |
| ELFN1 | 0.1514500069277972 | 0.0010657835266108 | 0.133496029139334 | 0.0003997541633306 |
| EPHB3 | 0.2943651702648178 | 8.428650578774194e-06 | 0.4197841032112299 | 0.0010196730485076 |
| FAM174B | 0.3397035818153082 | 0.0091055825700311 | 0.3403101546082726 | 0.0115482785534575 |
| GABRR2 | 0.3701014322548088 | 0.0013393020759646 | 0.3734295070283174 | 0.0012370192335172 |
| GOLGA7B | 0.2281767474527535 | 1.7584894020625757e-06 | 0.2294656771453861 | 1.321099647701833e-06 |
| GPR146 | 0.3034709404665114 | 9.599327424260096e-06 | 0.4548006651306834 | 0.0007560914256317 |
| GRIA4 | 0.4484351990666543 | 0.017294336202332 | 0.375705726076395 | 0.0051943126126698 |
| HABP4 | 2.805675230118376 | 4.3433956678407776e-106 | 2.139839599907336 | 2.8048467317345673e-55 |
| HOMER3-AS1 | 22.349170090955635 | 0.013384429286879 | 27.628614450998462 | 0.004395738036635 |
| HPCAL4 | 0.1592742997623776 | 5.1353660011712406e-05 | 0.2510678203002816 | 0.0005127636319964 |
| IGFN1 | 0.2568075396142625 | 3.9779479854828034e-12 | 0.314739176342406 | 1.5766388922496732e-10 |
| IRS4 | 0.2732823312367881 | 0.0009021221894898 | 0.3669035350830066 | 0.0050950851545332 |
| ITGA7 | 0.2942372634183782 | 1.759879857247586e-06 | 0.492921173181988 | 0.0011187675955634 |
| LIPG | 0.4013377113014887 | 7.524680053272731e-13 | 0.3468319121332001 | 6.576205316912995e-17 |
| LOC101927476 | 0.3829485068993029 | 0.0173706353394575 | 0.2068877750562044 | 0.0001972599922286 |
| LOC105373063 | 0.4822683402540312 | 0.0112854115411425 | 0.4459272594943105 | 0.009995130086392 |
| LOC105373690 | 0.3655479203164877 | 0.007423600333527 | 0.2422519526880588 | 0.000417250448955 |
| MAT1A | 0.4703770630963766 | 0.0070219462236713 | 0.4759529982483123 | 0.0098873219578265 |
| MMP9 | 0.1775024308720865 | 1.0278799048571452e-05 | 0.2096508585218157 | 4.669539691660957e-05 |
| NGFR | 0.3239967527922314 | 8.016235968722622e-16 | 0.383054163353035 | 5.344066995718649e-13 |
| NRXN2 | 0.0446256048687791 | 0.0027339784562586 | 0.0230638977425332 | 0.0003270170108736 |
| NXPH4 | 0.2341191715424568 | 2.87510665522799e-13 | 0.4681204074269736 | 5.5574037861726265e-06 |
| PCDH1 | 0.1871249878799802 | 0.0006223366722443 | 0.2744234969856189 | 0.0032103182724272 |
| PDE4A | 0.4053311541850781 | 0.0004908635352576 | 0.4186742635195875 | 0.0002972769284076 |
| PHOSPHO1 | 0.2896450351414701 | 5.035915048531913e-06 | 0.4803706021537285 | 0.0019262495305061 |
| PPFIA4 | 0.3090867843041712 | 8.980607303035737e-35 | 0.4957954414974198 | 2.4942981174798504e-16 |
| PRELID2 | 0.3989531264135992 | 6.626260418282526e-35 | 0.4756923318675632 | 1.2942055121043309e-30 |
| RAMACL | 0.3055316122051005 | 0.0038845775386953 | 0.2772852471896689 | 0.0018495571734986 |
| RASGRP2 | 0.4445333901115117 | 0.0026720753518237 | 0.4859110224068347 | 0.0079435367357372 |
| RIMKLA | 0.3241543310989747 | 1.3272533090018061e-107 | 0.4569467531009646 | 1.060353449510009e-56 |
| RND2 | 0.3745250354449795 | 0.001096300285868 | 0.4743021462791338 | 0.0080679428876861 |
| RNF152 | 0.4127881380570418 | 0.0058131928287697 | 0.4656197562070865 | 0.0140485890917973 |
| RORA | 0.1669442130887554 | 3.6611402178294683e-08 | 0.3308361776291462 | 2.9311403019081355e-05 |
| RUFY1-AS1 | 0.181738008124082 | 0.0032442851425261 | 0.2970457937443252 | 0.014036291198135 |
| SCARF1 | 0.3747779941448342 | 0.0001669320911474 | 0.2921982054262057 | 4.574278821975785e-07 |
| SEMA3F | 0.2996498768346066 | 3.270005160735356e-09 | 0.4095769995145746 | 3.000597539757157e-06 |
| SEMA7A | 0.4811977576975033 | 1.2446530682961294e-05 | 0.4876210775647676 | 1.4984308710456454e-05 |
| SETD7 | 2.6394883451903017 | 3.4666838251867915e-135 | 2.120522614835416 | 2.2116643059499963e-92 |
| SH2D3C | 0.1698420113650886 | 5.685482342877389e-06 | 0.4069999270572741 | 0.0052906790195809 |
| SLC4A8 | 0.1343451301568023 | 6.532942347728332e-08 | 0.1775408797515776 | 7.662108694497964e-07 |
| SMC5-DT | 2.4039854422264924 | 0.0160742077026955 | 2.553504793541889 | 0.0087054172029955 |
| SMIM11 | 0.130038930690066 | 0.000102096210492 | 0.207427387641656 | 0.0006813532708083 |
| SP2-AS1 | 2.5862280165852534 | 1.3461405490302995e-05 | 2.0568100519324304 | 0.0008593949496713 |
| TNFRSF19 | 0.3876109306420655 | 1.7030509408890244e-05 | 0.4565569923444996 | 0.0008892822961253 |
| TNFRSF9 | 0.2844839983373828 | 6.238667688992639e-05 | 0.2193576137731363 | 1.1379086174078637e-06 |
| UNC5B | 0.2222088662090574 | 0.0006954138324712 | 0.2118103838779347 | 0.0010013129702439 |
| VLDLR | 0.4228839778902639 | 2.2550703488761113e-22 | 0.4079529791142556 | 7.305211172615459e-23 |
| VWA5A | 0.4149072556836126 | 0.0088054874132862 | 0.3587262681148815 | 0.001389382016046 |
| VWA5B2 | 0.2281397283168585 | 1.950374067494931e-06 | 0.4513457289709978 | 0.0025093572081923 |
| ZDHHC11B | 0.1952058532721202 | 1.8584334204312664e-06 | 0.3400549851595968 | 0.0004022321906865 |
| ZNF521 | 0.0489877049760935 | 0.0041316406934016 | 0.0493045632951915 | 0.0040333368458212 |
| ZNF616 | 2.008174985978433 | 8.747996725635299e-13 | 2.1361038196460167 | 1.5701465733864564e-18 |
| ZNF687-AS1 | 2.2957572421254957 | 0.0010970989284981 | 2.0889002543952557 | 0.003531285313962 |
| ZSWIM9 | 2.035102789811909 | 1.3216940893791193e-18 | 2.0735999488436856 | 2.3023777085745585e-19 |

**Supplementary Materials and Methods**

**Antibodies and reagents**

Anti-USP 13 antibody (Cat. No. 16840-1-AP), anti-PCMT1 antibody (Cat. No. 10519-1-AP), anti-AKT antibody (Cat No. 10176-2-AP), anti- Phospho-AKT (Ser473) antibody (Cat No. 66444-1-Ig), anti-GSK3β antibody (Cat No. 51065-1-AP), anti-STING antibody (Cat No. 19851-1-AP), anti-TBK1 antibody (Cat No. 83686-3-RR), anti-beta-actin antibody (Cat. No. HRP-66009), anti-HA antibody (Cat. No. HRP-81290), anti-Flag antibody (Cat. No. 66008-4-IG), and anti-Myc antibody (Cat. No. 16286-1-AP) were obtained from Proteintech (Wuhan, China). Anti-Ki67 antibody (ab 15580) was obtained from Abcam (Cambridge, United Kingdom). Goat anti-mouse IgG secondary antibody (#7076) and goat anti-rabbit IgG secondary antibody (#7074) were purchased from Cell Signaling Technology (Danvers, USA). Alexa Fluor 488 labeled goat anti-mouse IgG (H+L) and Cy3 labeled goat anti-mouse IgG (H+L) were purchased from Beyotime Biotechnology (Shanghai, China). All antibodies were used according to the manufacturer's guidelines.

**Plasmids and cloning**

Myc-USP13 (P63352), Myc-USP13 (1-300aa), Myc-USP13 (301-625aa), Myc-USP13 (626-863aa) and Flag-PCMT1 (P53692) expression vectors were constructed by subcloning into the pcDNA3 plasmid and were sourced from MiaoLingBio, China. USP13 mutant was generated by using the QuikChange II Site-Directed Mutagenesis Kit. Primers used for cloning are available upon request

**shRNA lentiviral vector packaging and transduction**

shRNA lentiviral vector packaging and transduction were described as previously. lentiviral vectors shRNA-USP13 and shRNA-GFP pLKO1 (control vector) were purchased from QEgene (Shanghai, China) . Lentiviral vector encoding shRNA was packaged in 293T cells by calcium phosphate transfection. The supernatants that contained lentiviral particles were collected 48h after transfection. The indicated PCa cells were then transduced with the supernatant in the presence of polybrene (8 μg/mL) for 24 h before replacement with fresh growth media. Cells were analyzed at 48 or 72 h post transduction.

**Quantitative real-time PCR (qRT-PCR)**

qRT-PCR was described as previously. Primers for qPCR analysis of human gene transcripts were:

USP13:

Forward Primer: 5’-CCTCTAACAGGCAGCAAGATGC-3’

Reverse Primer: 5’-GCACTGAATGCGTTCTTCCACC-3’

USP13-△UBP:

Forward Primer: 5’-ATGCAGCGCCGGGGCGCC-3’

Reverse Primer: 5’-CCCATGCATATGAAGCATATCA-3’

USP13-△USP:

Forward Primer: 5’-GGGACAGAGAATGGGCTCCA-3’

Reverse Primer: 5’-TTCCTCTCCTGGCTGTAACC-3’

USP13-△UBA:

Forward Primer: 5’-GAAGAACTTCCAGACATCAGCC-3’

Reverse Primer: 5’-GCTTGGTATCCTGCGGTAAAAG-3’

β-actin：

Forward Primer: 5’-TCTCCCAAGTCCACACAGG-3’

Reverse Primer: 5’-GGCACGAAGGCTCATCA-3’

PCMT1：

Forward Primer: 5’-GAAGTGATGCTGGCTACAGACC-3’

Reverse Primer: 5’-ATGTGTGGAGCACTGATTGTTGC-3’

PSA:

Forward Primer: 5’- CACCTGCTCGGGTGATTCTG-3’

Reverse Primer: 5’- CCACTTCCGGTAATGCACCA -3’

NKX3.1:

Forward Primer: 5’- ACTTGGGGTCTTATCTGTTGGA -3’

Reverse Primer: 5’- CTCGATCACCTGAGTGTGGG -3’

FKBP5:

Forward Primer: 5’- AATGGTGAGGAAACGCCGATG -3’

Reverse Primer: 5’- TCGAGGGAATTTTAGGGAGACT-3’

KLK2:

Forward Primer: 5’- TCAGAGCCTGCCAAGATCAC -3’

Reverse Primer: 5’- CACAAGTGTCTTTACCACCTGT-3’

Western blotting

Proteins were prepared as previously described[^1^](#_ENREF_1). Briefly, protein extracts were separated by gradient SDS-PAGE gel and then electroblotted onto a PVDF membrane (Cytiva, catalog number: 10600021). The membranes were incubated with the indicated primary antibodies at 1: 1000 at 4°C overnight, respectively, followed by incubation with corresponding secondary antibodies at 1: 10000 at room temperature for 1 h.

Cell proliferation and clony formation assays

Cell proliferation and clony formation were described as previously [^2^](#_ENREF_2).

**Wound healing**

To assess the migration capacity of PCa cells, designated PCa cells were seeded in triplicate onto 6-well plates and incubated overnight at 37℃ and 5% CO^2^ for 24 hours. When the cells reached more than 90% confluence, linear scratches were made along the length of each well using a 200 µL pipette tip. Cells were then maintained in medium with reduced serum (3% FBS). Plates were imaged using Celigo Imaging Cytometer at indicated time points. Each image was quantified using ImageJ. The uncovered area for each well was calculated as the average percentage of the three wells and expressed as mean ± SD. This experiment represents three experiments.

**Edu assay**

Edu assays were performed as described previously[^2^](#_ENREF_2).

**Migration and invasion**

Transwell assays were performed using 24-well plates equipped with 8 μm polycarbonate membrane filters (Corning Inc., Corning, NY), either coated with or without Matrigel. Designated PCa cells (1 x 10^4^) were seeded in the top wells of serum-free 1640 or DMEM medium while 700.mu.l of medium supplemented with 10% FBS was added to the lower chamber as chemoattractant. After incubation for 24-48 hours, cells were fixed with 4% paraformaldehyde for 15 minutes and stained with 0.1% crystal violet for 10 minutes. Cells underneath the inserts were analyzed using light microscopy and photographed. The numbers of cells were counted in five random fields for each insert. Each data point represents the average number from three wells.

**Protein immunoprecipitation (IP) and Liquid Chromatography-MS Analysis**

Co-IP or IP was described as previously [^3^](#_ENREF_3). In brief, cells were collected in RIPA lysis buffer, incubated on ice for 30 minutes, and then centrifuged at 12,000×g for 20 minutes. Cell lysates were immunoprecipitated with specific antibodies and incubated overnight at 4 ℃ with Flag-M2 beads or protein A/G agarose beads (Santa Cruz, USA), rotating gently. The beads were washed three times with lysis buffer. The eluted proteins were then separated using SDS-PAGE and analyzed by Western blotting. For Liquid Chromatography-MS Analysis, immunoprecipitation of USP13 antibodies was performed as described above. The precipitated proteins were eluted 3 times with lysis buffer. The eluted samples were subjected to in-solution trypsin digestion, followed by liquid chromatography-MS analysis and Protein identification was performed as previously described [^3^](#_ENREF_3).

Ubiquitination assay

Ubiquitination assay was described as previously[^4^](#_ENREF_4). Cells were transfected with the specified plasmids and lysed using immunoprecipitation buffer. During the immunoprecipitation process, 2 mg of protein was mixed with the designated antibodies and incubated overnight at 4 °C, after which Flag-M2 beads or protein A/G beads were added for 2 hours. The beads were washed once with TBS containing 1% Triton X-100 and 1% SDS, followed by two washes with 0.5 M LiCl and TBS buffer, and finally washed in PBS with 1% Triton X-100. Proteins were loaded onto 8% SDS-PAGE gels and immunoblotted with the indicated antibodies.

**Immunostaining**

Cultured PCa cells were fixed in 4% paraformaldehyde for 30 minutes and then permeabilized with 0.1% Triton X-100 in PBS for 5 minutes. After washing twice with PBS, the cells were blocked in PBS containing 2% BSA and 1% normal goat serum for 1 hour at room temperature. After washing again, the slides were incubated overnight with the primary antibody in blocking solution in a humidity chamber at 4℃. The following day, after three washes with PBS, the Alexa Fluor Cy 3-conjugated and/or Alexa Fluor 488-conjugated secondary antibodies were treated in blocking solution at 37℃. C. for 1 hour. the slide were then washed again three time with PBS and counterstained by 4, 6-diamidine-2-pheny-lindole (DAPI) for nuclear visualization. Images were captured using an inverted microscope with a ×40 objective and scanned with a laser confocal system.

**Luciferase assay**

The luciferase reporter gene constructs (promoter region of USP13 from the transcription start site 2000bp ) were transfected into the enzalutamide-resistant C4-2B cells for 48 h, which stably expressing shHIF1α. Firefly luciferase activity was used to normalize the Renilla luciferase activity for each transfected well (n = 3). The pRL-TK Renilla luciferase reporter vector was used as an internal control reporter vector (Promega). Luciferase activity was measured using the dual-luciferase reporter assay system (Promega). Renilla luciferase activity was used to normalize the firefly luciferase activity (n = 3).

pGL3.0-promoter:

Forward Primer: cgagctcttacgcgtgctagcGCACCCACATGGGAGACCC

Reverse Primer: acttagatcgcagatctcgagCTCCAAAGATTTGTACAAGATGGG

**RNA-seq analysis**

Total RNA from the enzalutamide-resistant C4-2B cells with stable knockdown of USP13 (shUSP13), PCMT1 (shPCMT1), and non-targeting control (NC) were extracted and subjected to RNA sequencing with Illumina NovaSeq 6000 by Novogene Inc. (Shanghai, China). The transcriptome sequencing and analysis were conducted by OE Biotech Co., Ltd. (Shanghai, China). Differential expression analysis was performed using the DESeq2. Q value < 0.05 and fold change > 2 was set as the threshold for significantly differential expression gene (DEGs). Hierarchical cluster analysis of DEGs was performed using R (v 3.2.0) to demonstrate the expression pattern of genes in different groups and samples.

**PCa PDX models**

PCa patient tumor fragments (25-40 mm^3^) were digested into single cells and then the cells were infected virus of only USP13 knockdown and or combined PCMT1 overexpression plasmids. After one month, the proteins form the above the tumors were subjected to detect the indicated proteins as in the Fig. S6M by Western Blotting.

Supplemental References

1. Xu S, Fan L, Jeon HY, Zhang F, Cui X, Mickle MB*, et al.* p300-Mediated Acetylation of Histone Demethylase JMJD1A Prevents Its Degradation by Ubiquitin Ligase STUB1 and Enhances Its Activity in Prostate Cancer. *Cancer research* 2020, **80**(15)**:** 3074-3087.

2. Xiong S, Li S, Li Z, Song Y, Yang L, Yang H*, et al.* A noncanonical E3 ubiquitin ligase RNF41-mediated MYO1C stability promotes prostate cancer metastasis by inducing actin remodeling. *Oncogene* 2024, **43**(36)**:** 2696-2707.

3. Xiong Y, Dong Q, Hu H, Li Z, Zhan X, Zheng F*, et al.* Glucose-induced STUB1-GOT2 axis promotes aspartate synthesis and mitochondrial dysfunction in bladder cancer. *Cell death & disease* 2025, **16**(1)**:** 516.

4. Zheng F, Li S, Xiong S, Li Z, Yuan R, Wang Z*, et al.* Loss of RNF41 promotes bladder cancer metastasis through increasing NUDC stability to enhance tubulin polymerization. *Cell death & disease* 2025, **16**(1)**:** 443.
