# Supplementary material for: USP13 promotes enzalutamide resistance by catalyzing depolyubiquitination of PCMT1 in prostate cancer
Source: Cell Death Dis. 2026 Apr 30;17(1):576. doi: 10.1038/s41419-026-08824-9 (PMC13272797; doi:10.1038/s41419-026-08824-9)

Figure1

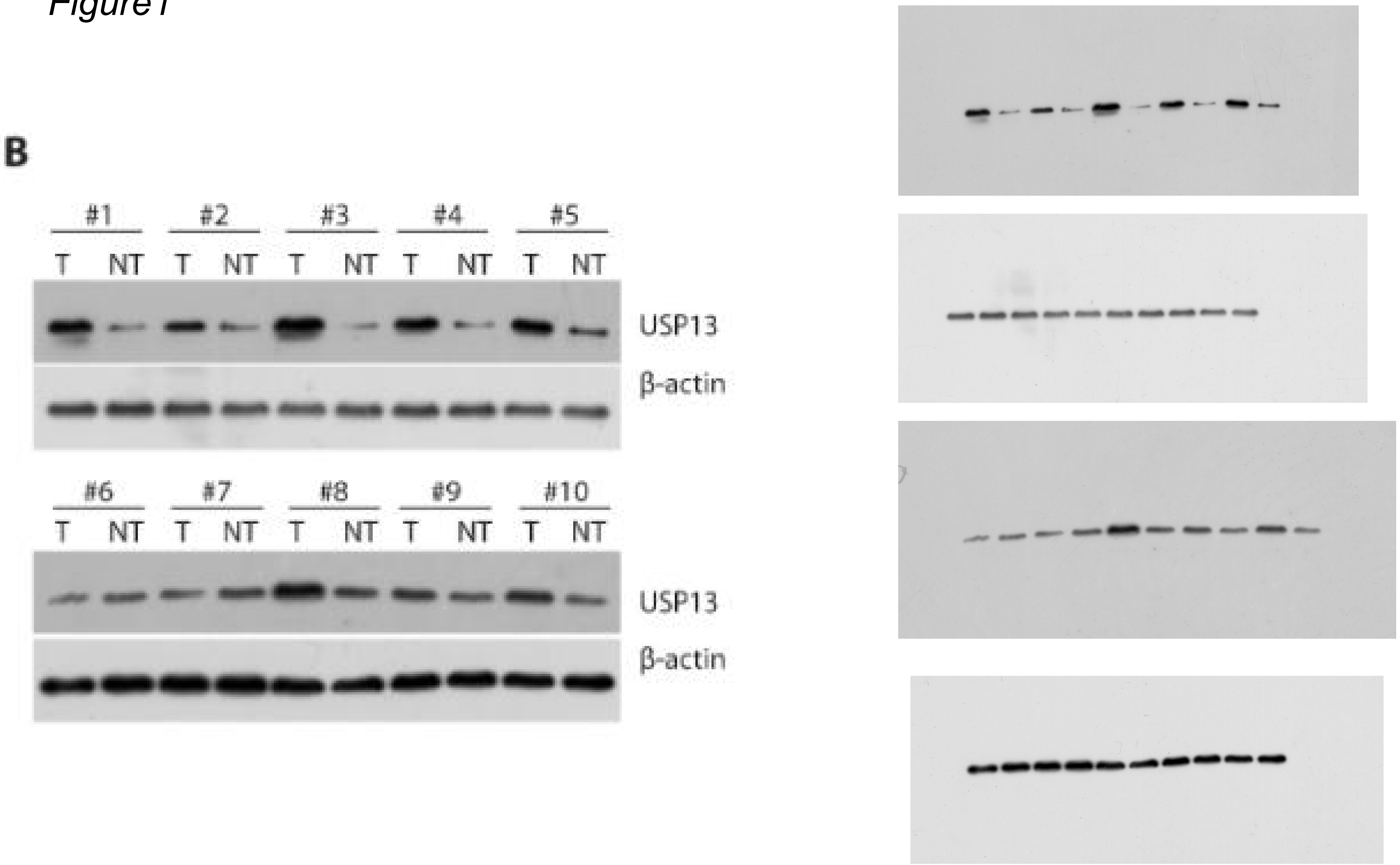
B
#1
#2
#3
#4
#5
T NT T NT T NT T NT T NT
USP13
β-actin
#6
#7
#8
#9
#10
T NT T NT T NT T NT T NT
USP13
β-actin

Figure2

C

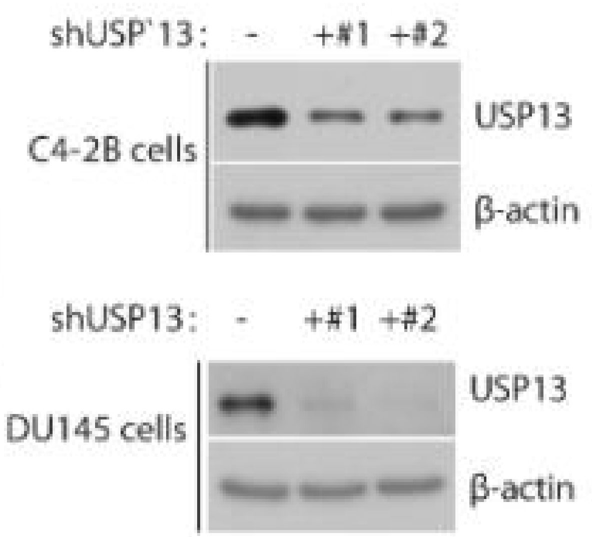
shUSP'13 : - +#1 +#2
C4-2B cells
USP13
β-actin
shUSP13 : - +#1 +#2
DU145 cells
USP13
β-actin

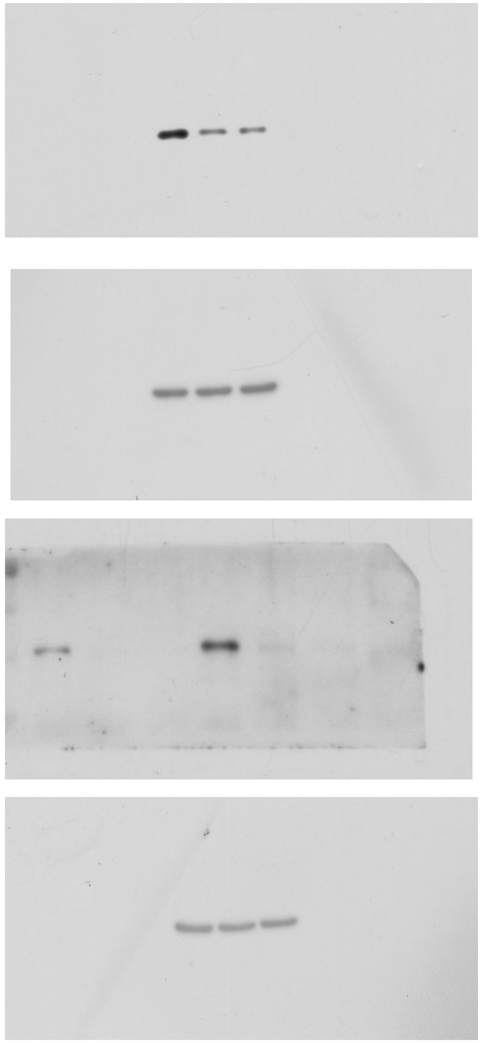

Figure3

E

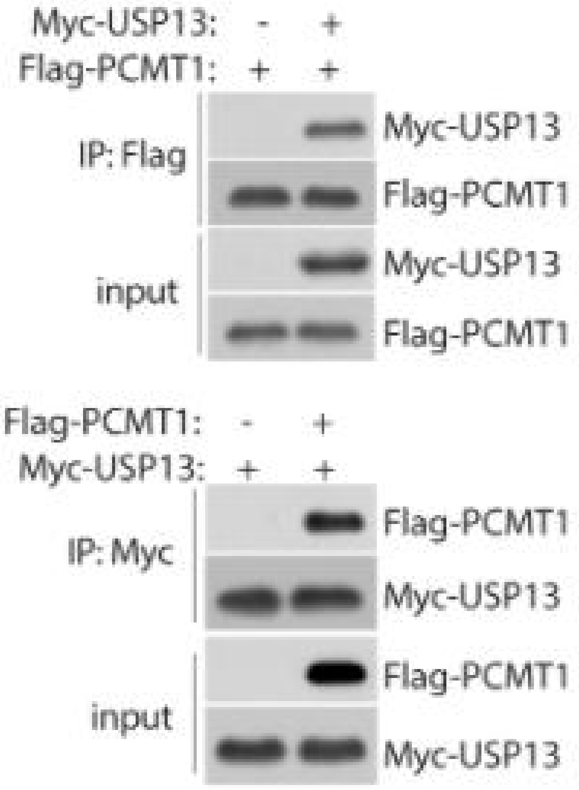
Myc-USP13: - +
Flag-PCMT1: + +
IP: Flag
Myc-USP13
Flag-PCMT1
input
Myc-USP13
Flag-PCMT1
Flag-PCMT1: - +
Myc-USP13: + +
IP: Myc
Flag-PCMT1
Myc-USP13
input
Flag-PCMT1
Myc-USP13

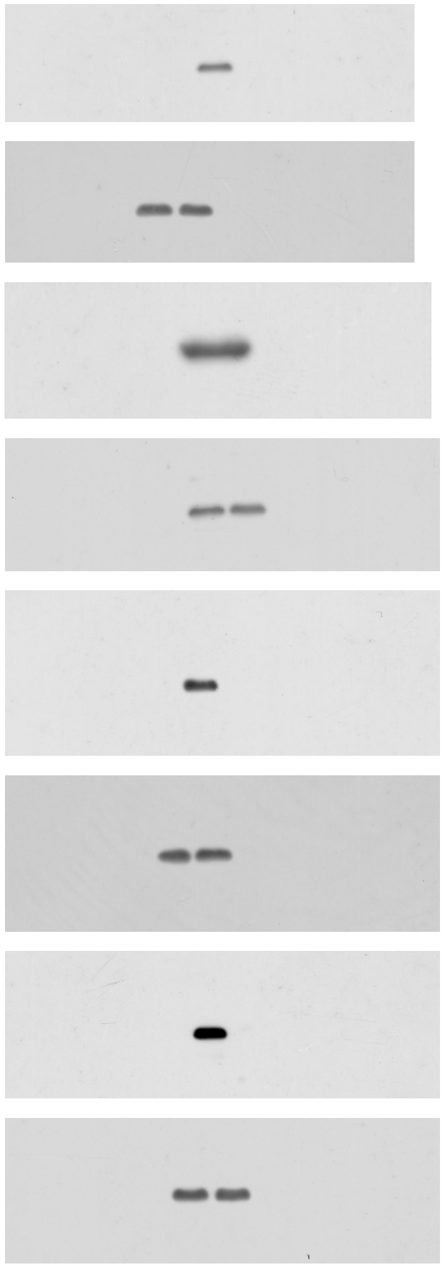

Figure3

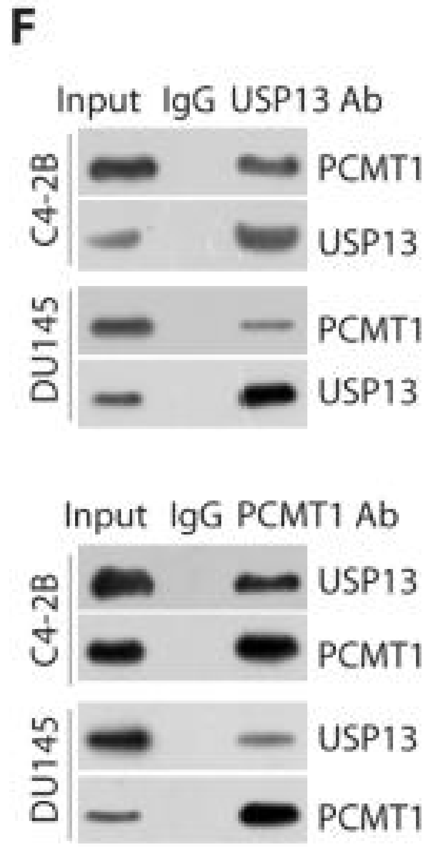
F
Input
IgG
USP13 Ab
C4-2B
PCMT1
USP13
DU145
PCMT1
USP13
Input
IgG
PCMT1 Ab
C4-2B
USP13
PCMT1
DU145
USP13
PCMT1

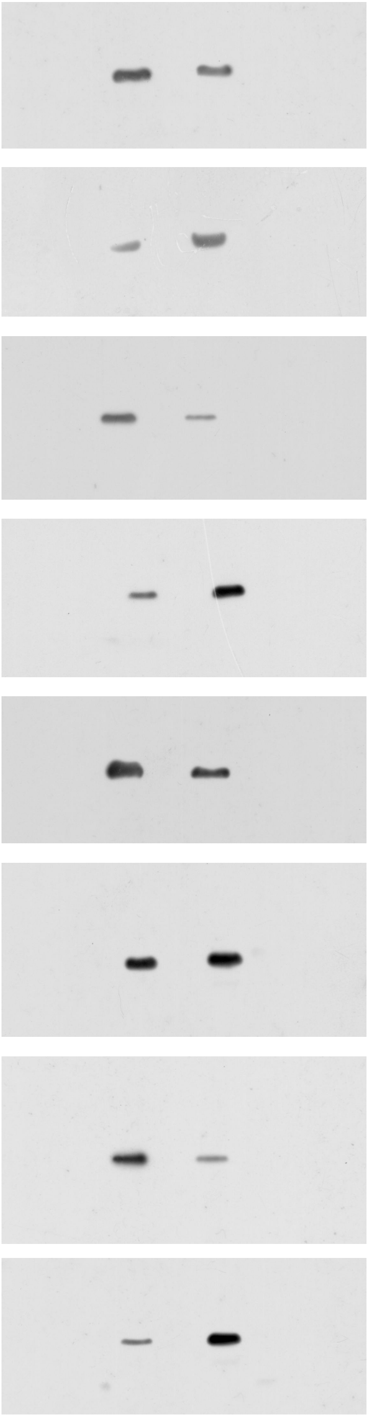

Figure3

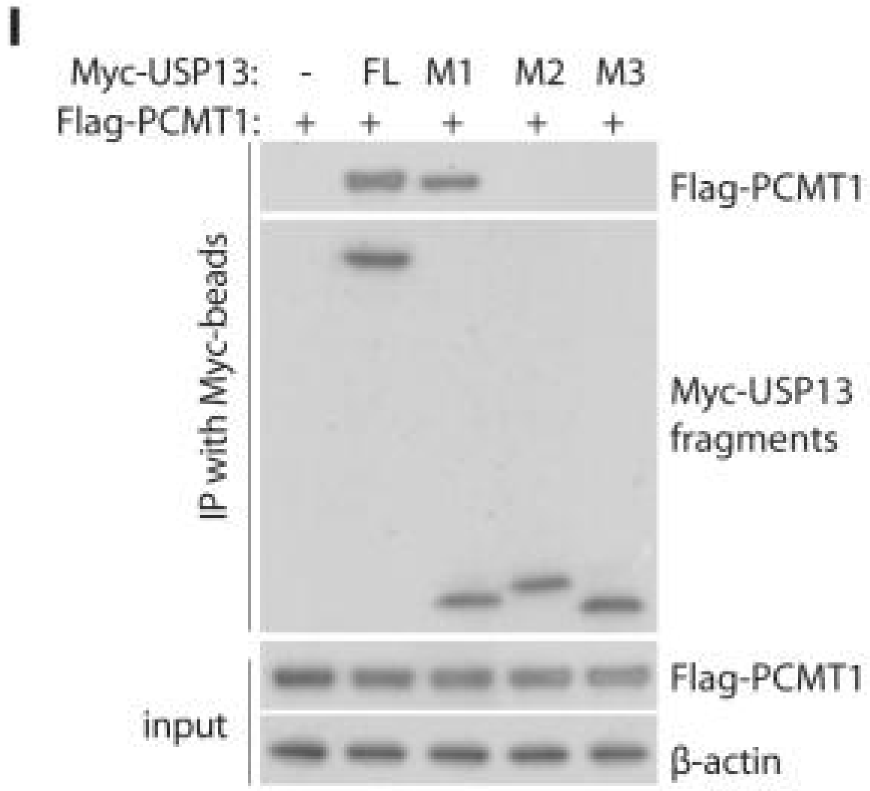
I
Myc-USP13: - FL M1 M2 M3
Flag-PCMT1: + + + + +
Flag-PCMT1
Myc-USP13 fragments
IP with Myc-beads
Flag-PCMT1
input
β-actin

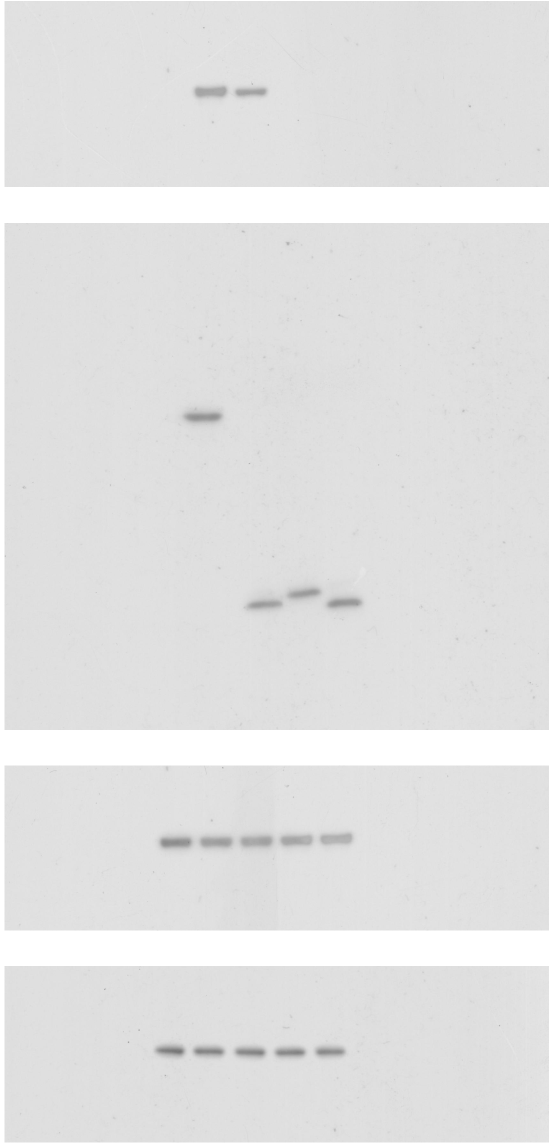

Figure4

A

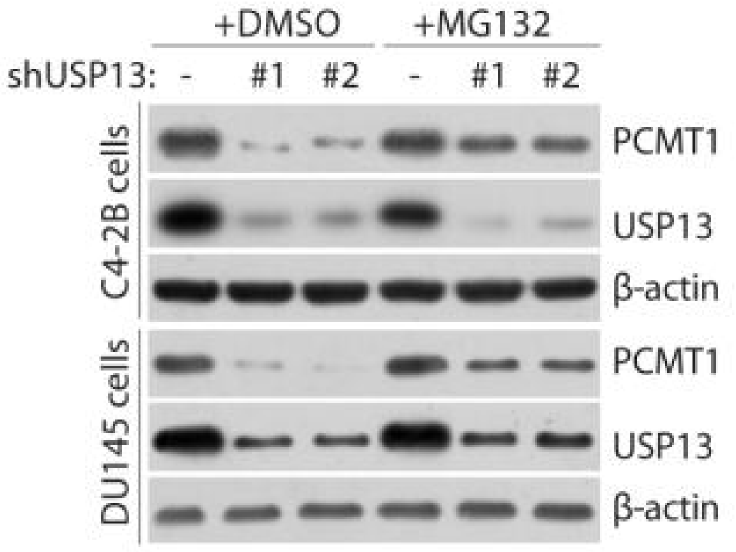
+DMSO
+MG132
shUSP13: - #1 #2 - #1 #2
C4-2B cells
DU145 cells
PCMT1
USP13
β-actin
PCMT1
USP13
β-actin

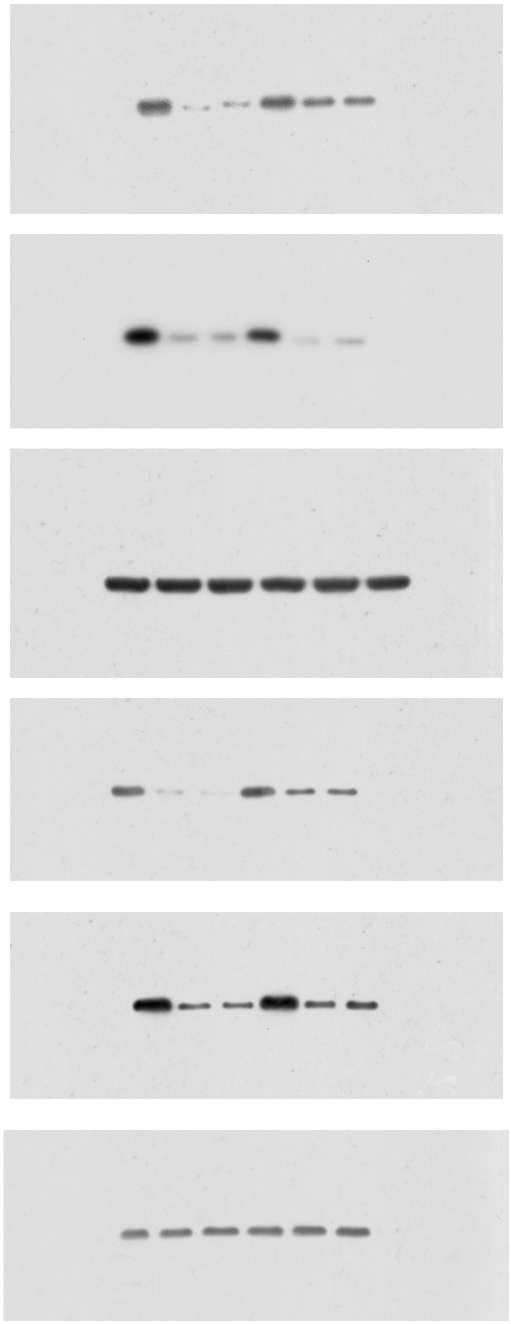

Figure4

B

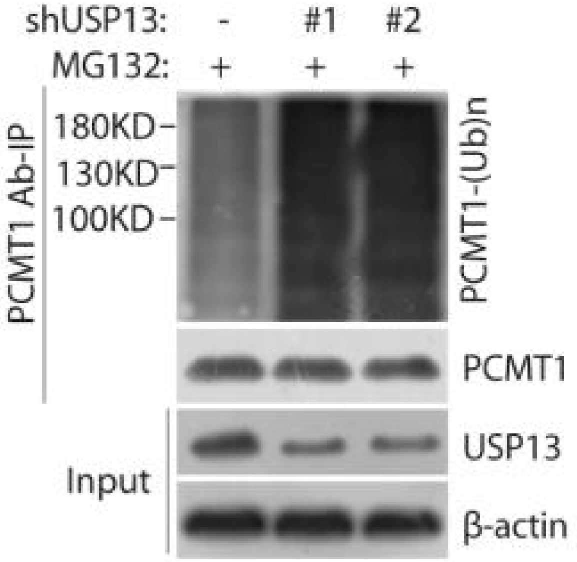

shUSP13: - #1 #2
MG132: + + +
PCMT1 Ab-IP
180KD
130KD
100KD
PCMT1-(Ub)n
PCMT1
USP13
Input
β-actin

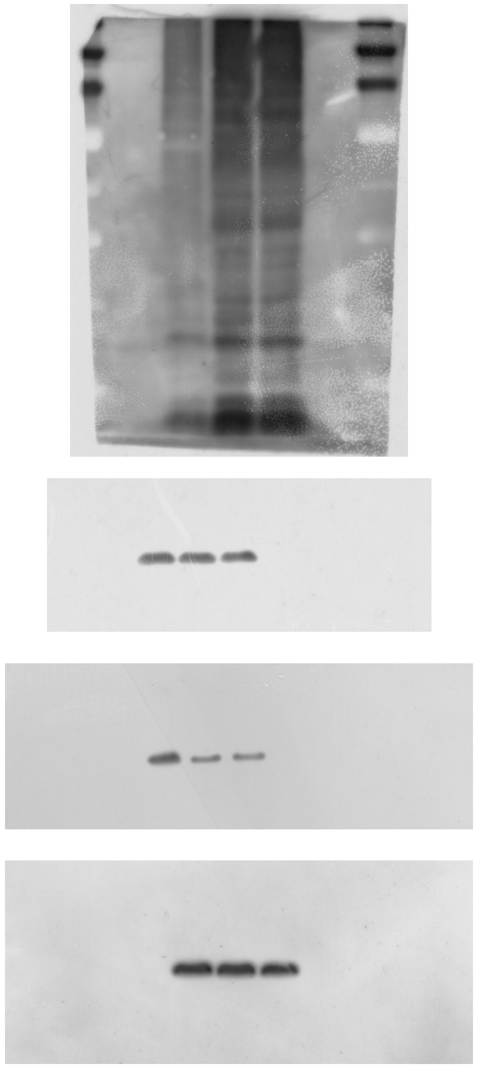

Figure4

C

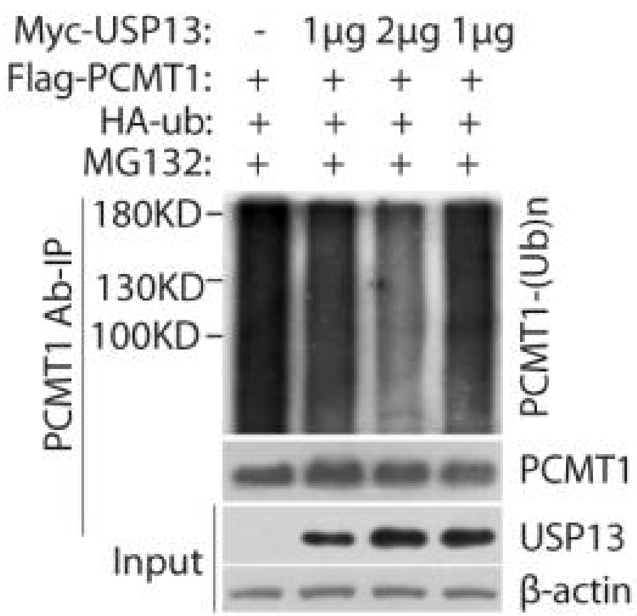

Myc-USP13: - 1μg 2μg 1μg
Flag-PCMT1: + + + +
HA-ub: + + + +
MG132: + + + +
PCMT1 Ab-IP
180KD
130KD
100KD
PCMT1-(Ub)n
PCMT1
Input
USP13
β-actin

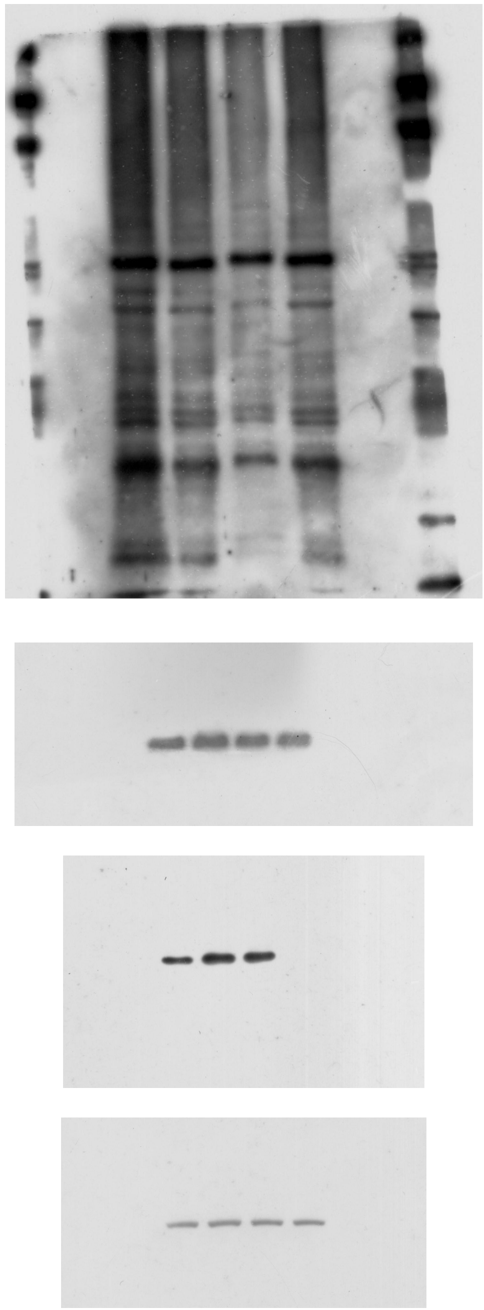

Figure4

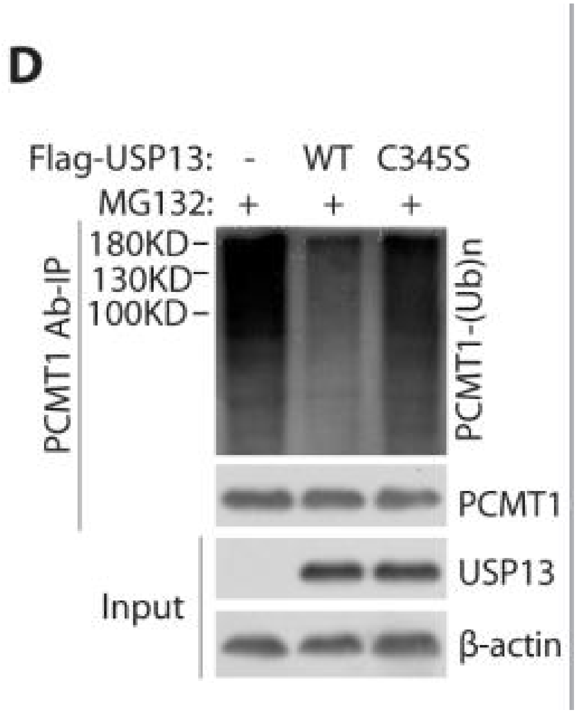

D
Flag-USP13: - WT C345S
MG132: + + +
180KD
130KD
100KD
PCMT1 Ab-IP
PCMT1-(Ub)n
PCMT1
USP13
Input
β-actin

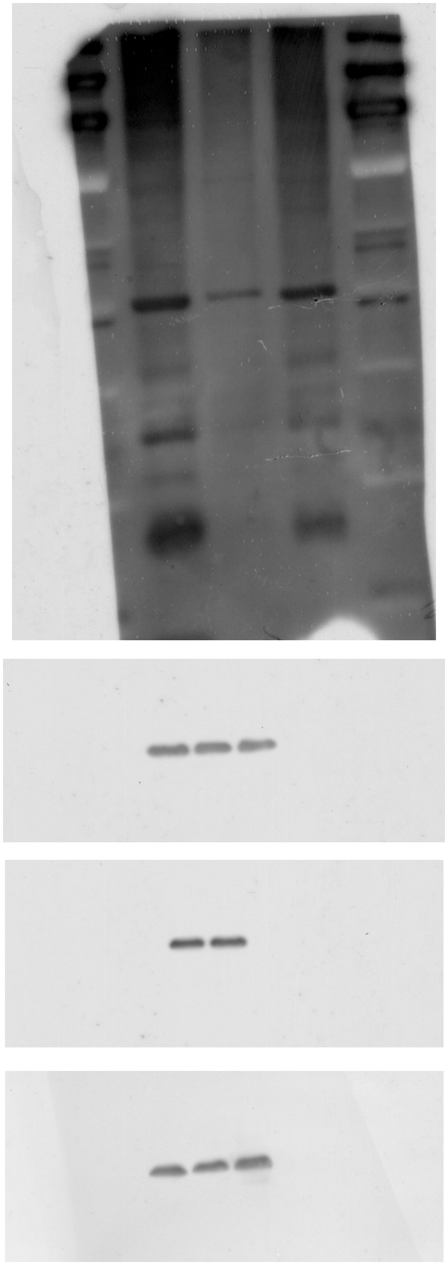

Figure4

E

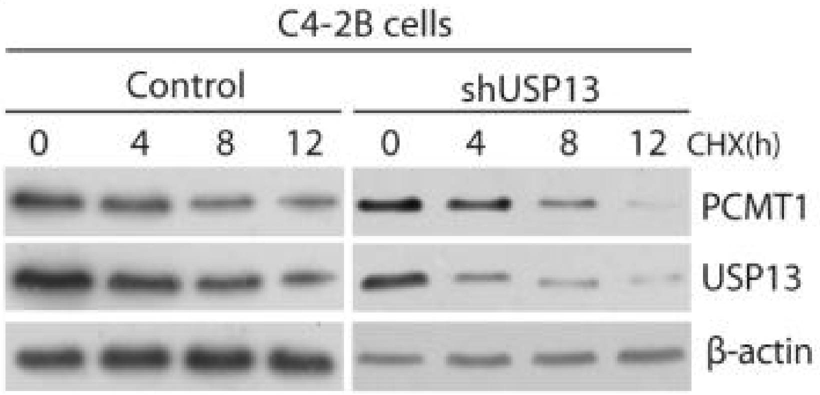
C4-2B cells
Control
shUSP13
0
4
8
12
0
4
8
12
CHX(h)
PCMT1
USP13
β-actin

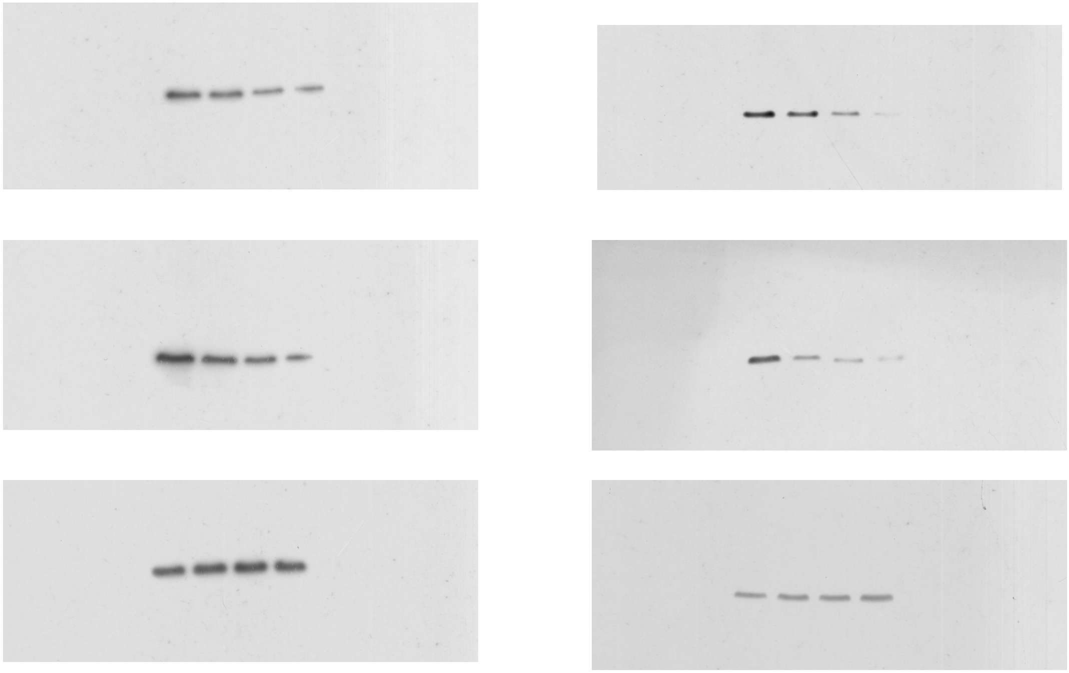

Figure4

F

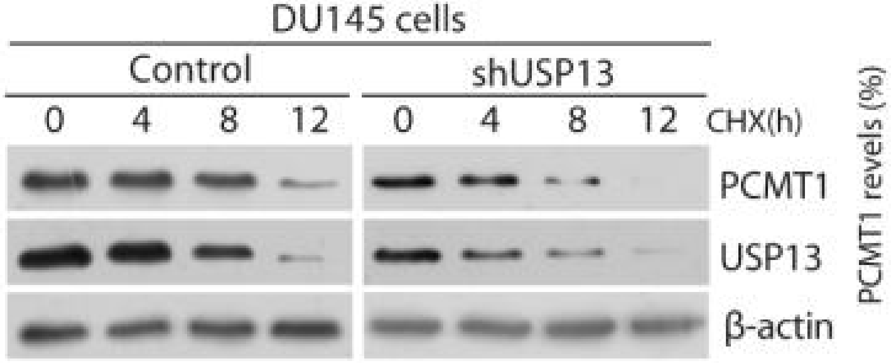

DU145 cells
Control
shUSP13
0
4
8
12
0
4
8
12
CHX(h)
PCMT1
USP13
β-actin
PCMT1 revels (%)

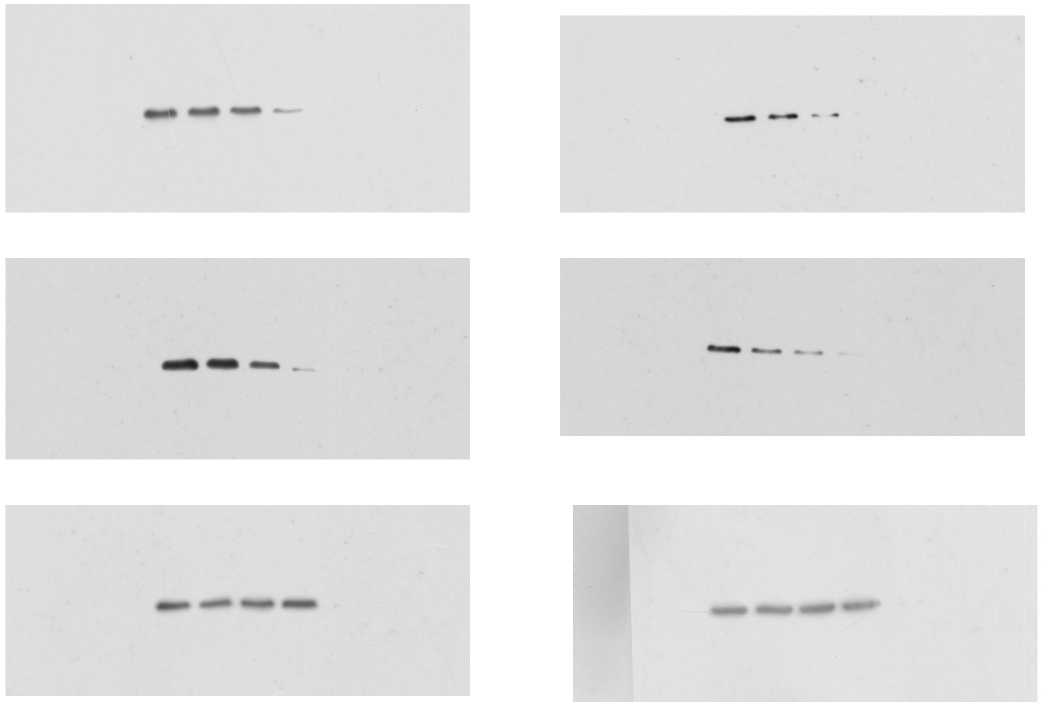

Figure4

G

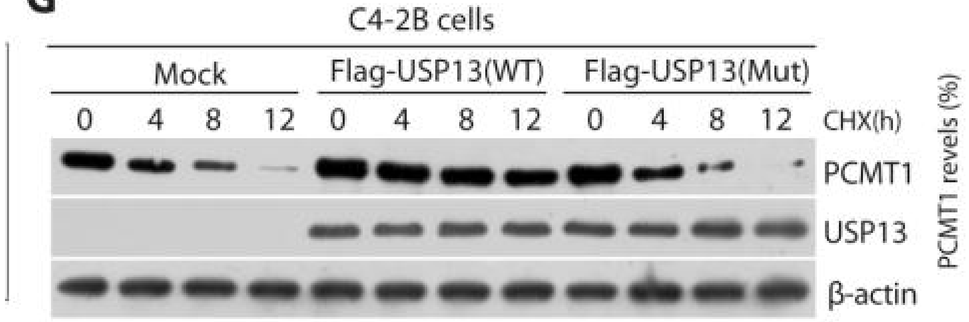
C4-2B cells
Mock
Flag-USP13(WT)
Flag-USP13(Mut)
0 4 8 12 0 4 8 12 0 4 8 12 CHX(h)
PCMT1
USP13
β-actin
PCMT1 revels (%)

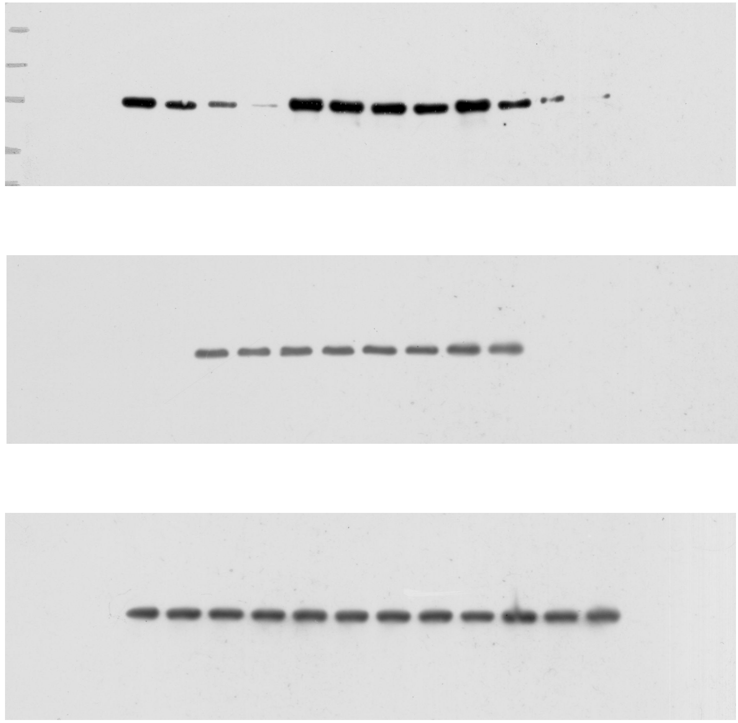

Figure4

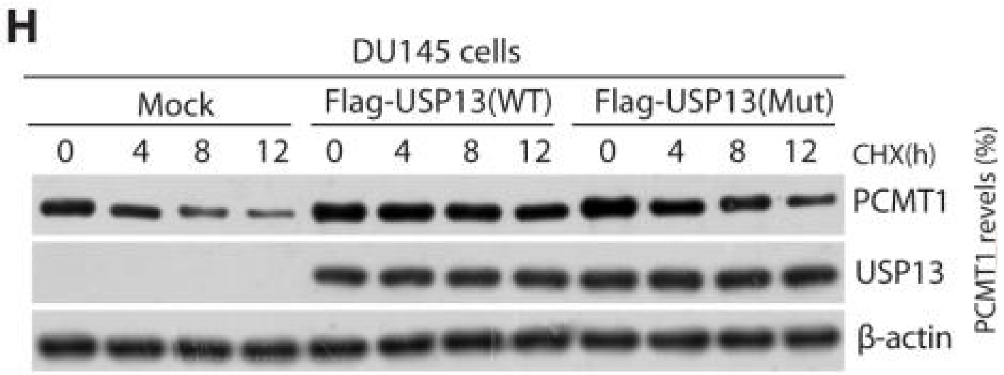
H
DU145 cells
Mock
Flag-USP13(WT)
Flag-USP13(Mut)
0
4
8
12
0
4
8
12
0
4
8
12
CHX(h)
PCMT1
USP13
β-actin
PCMT1 revels (%)

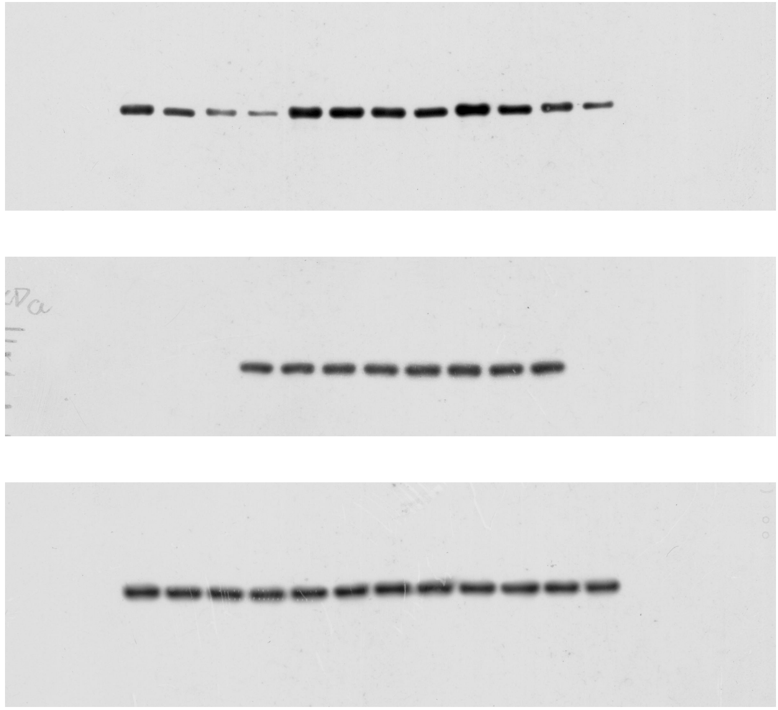

Figure5

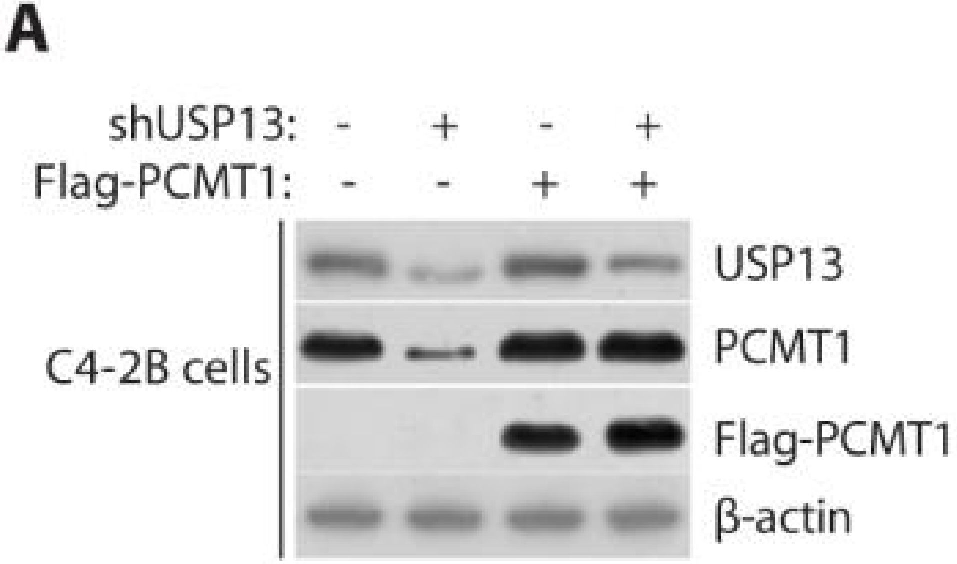

A
shUSP13: - + - +
Flag-PCMT1: - - + +
C4-2B cells
USP13
PCMT1
Flag-PCMT1
β-actin

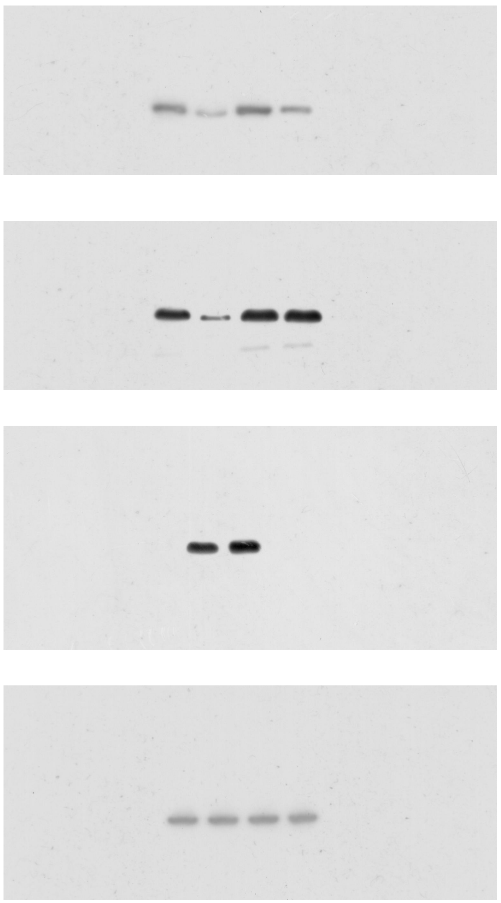

Figure5

B

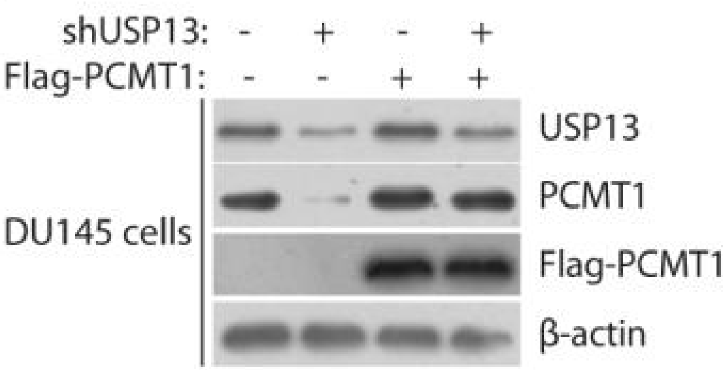

shUSP13: - + - +
Flag-PCMT1: - - + +
DU145 cells
USP13
PCMT1
Flag-PCMT1
β-actin

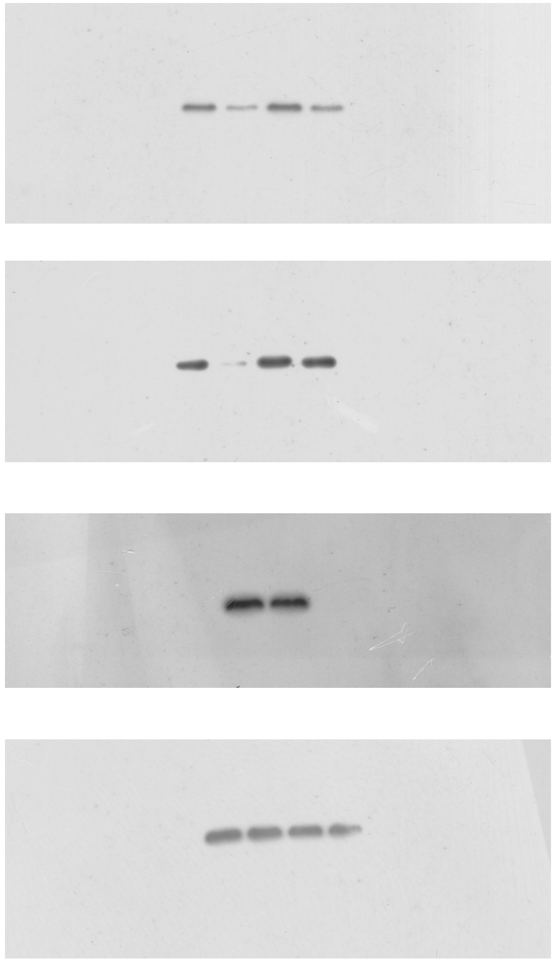

Figure6

D

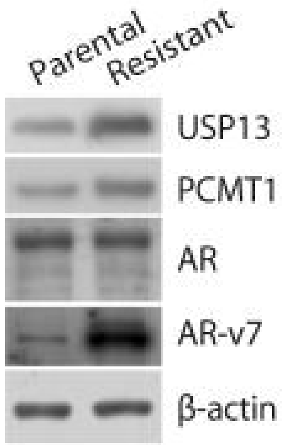

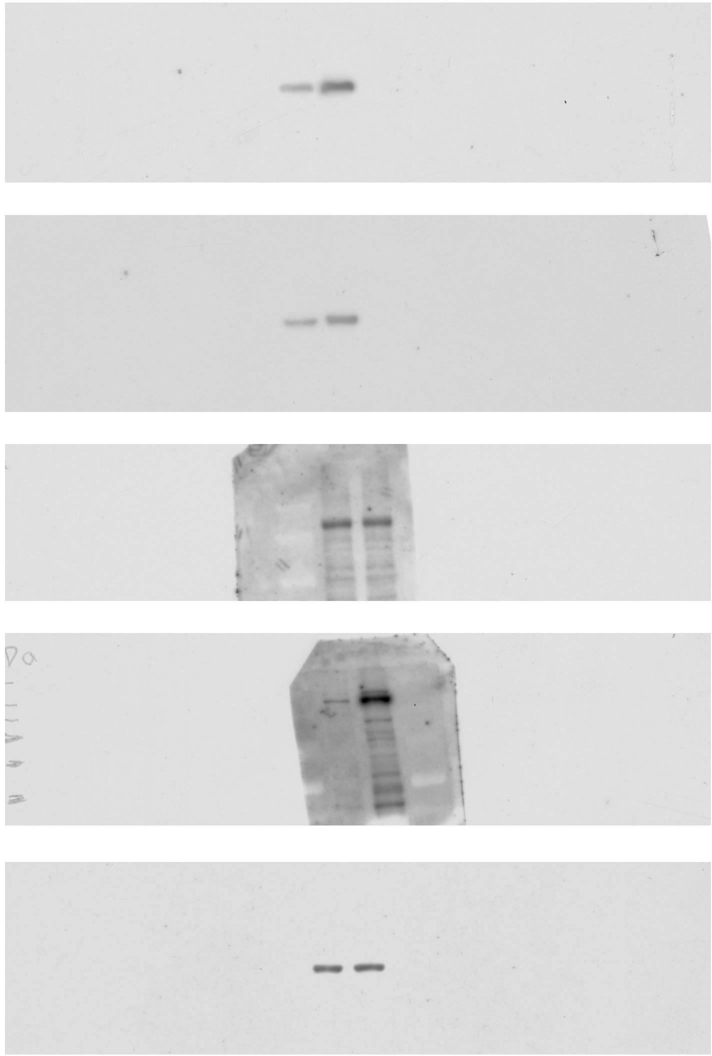

Figure6

F

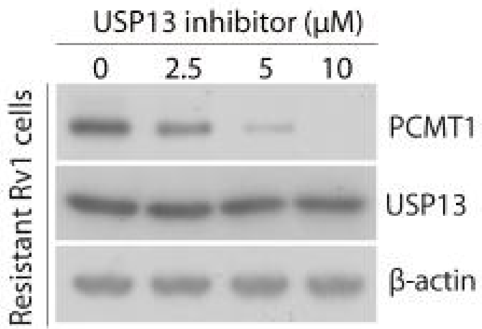
USP13 inhibitor (μM)
0
2.5
5
10
Resistant Rv1 cells
PCMT1
USP13
β-actin

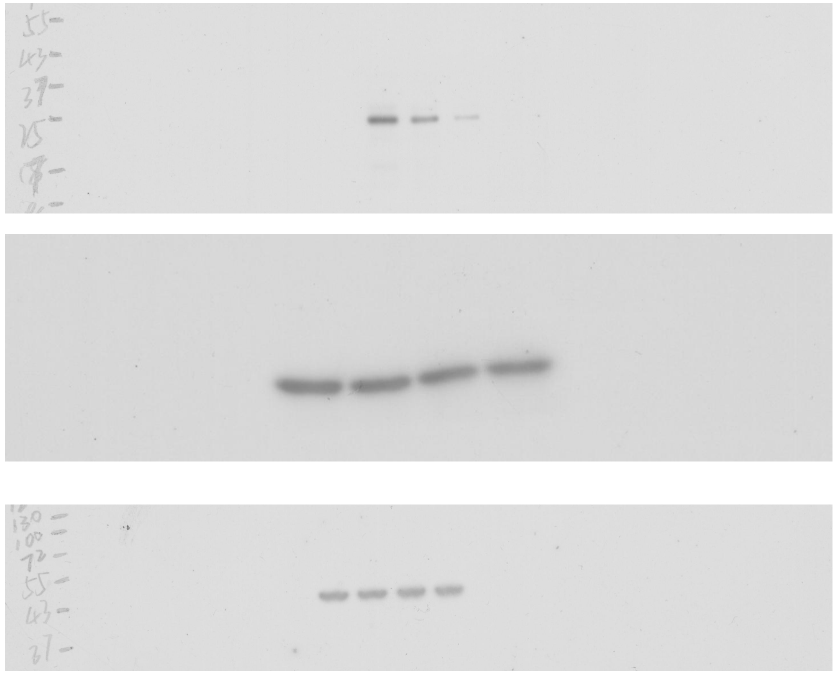

Figure6

G

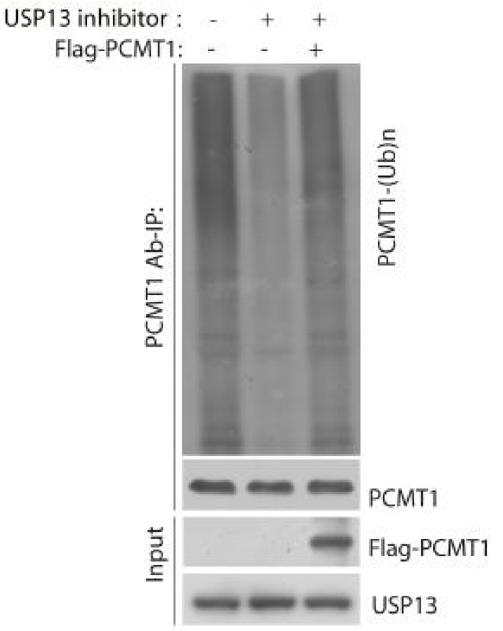

USP13 inhibitor : - + +
Flag-PCMT1: - - +
PCMT1 Ab-IP:
PCMT1-(Ub)n
PCMT1
Input
Flag-PCMT1
USP13

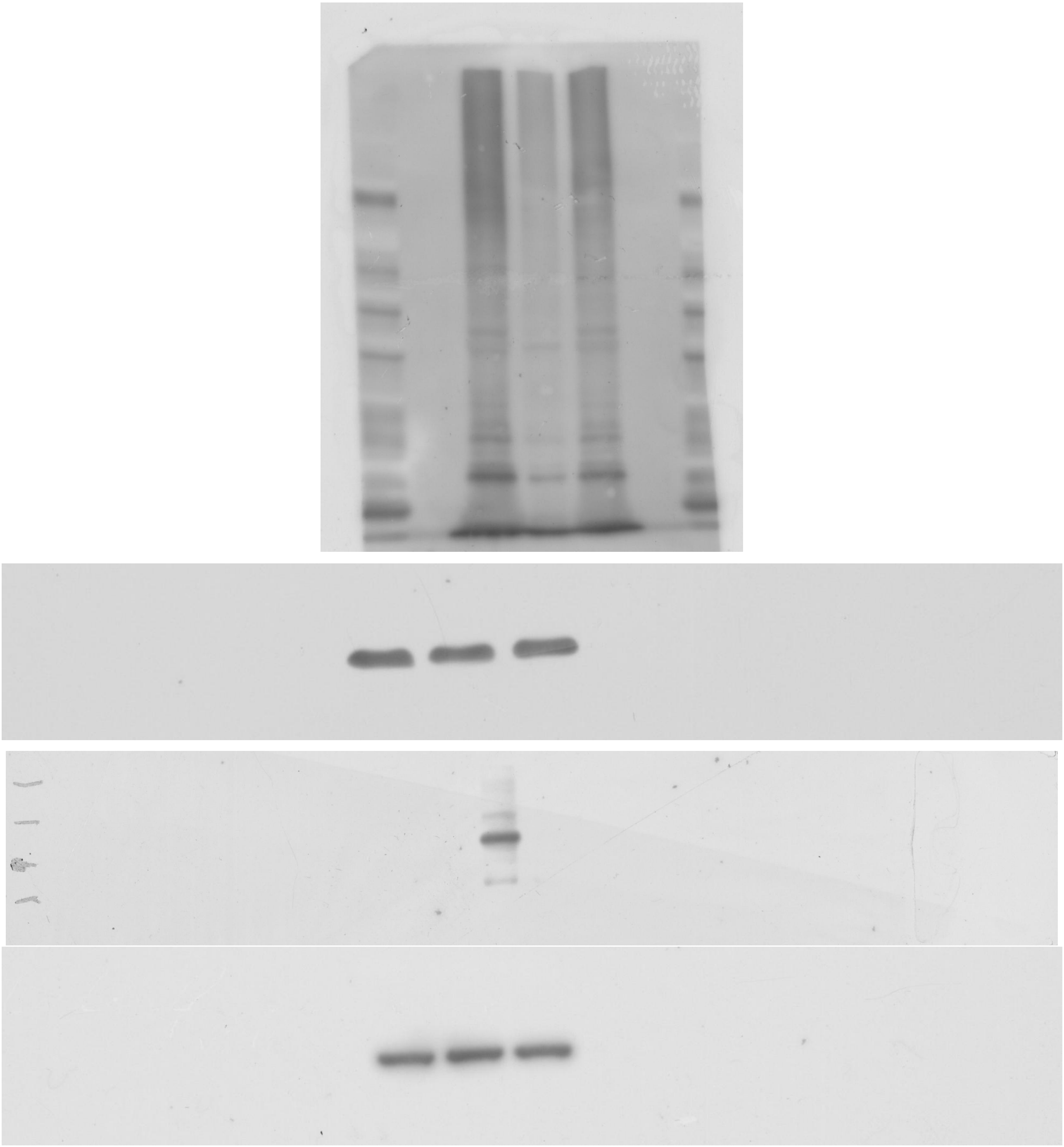

Figure6

L

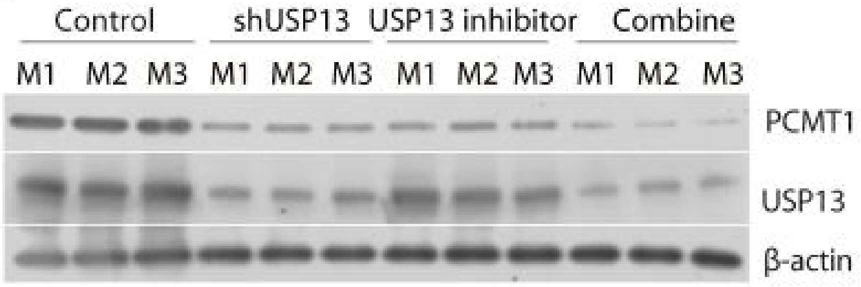

Control
shUSP13
USP13 inhibitor
Combine
M1 M2 M3 M1 M2 M3 M1 M2 M3 M1 M2 M3
PCMT1
USP13
β-actin

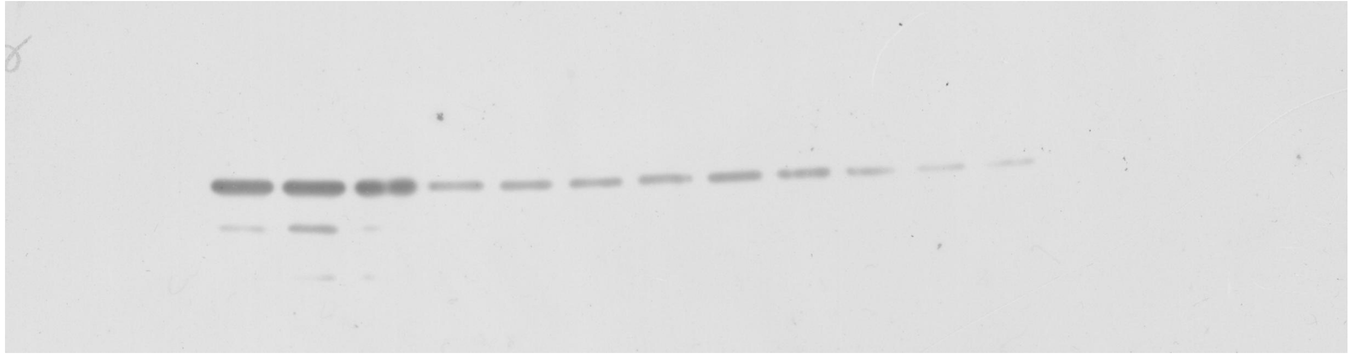

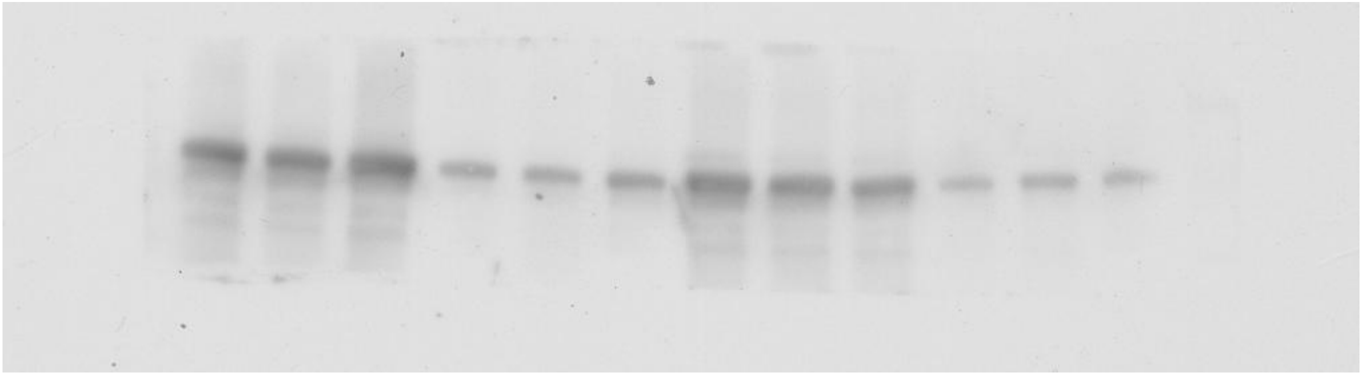

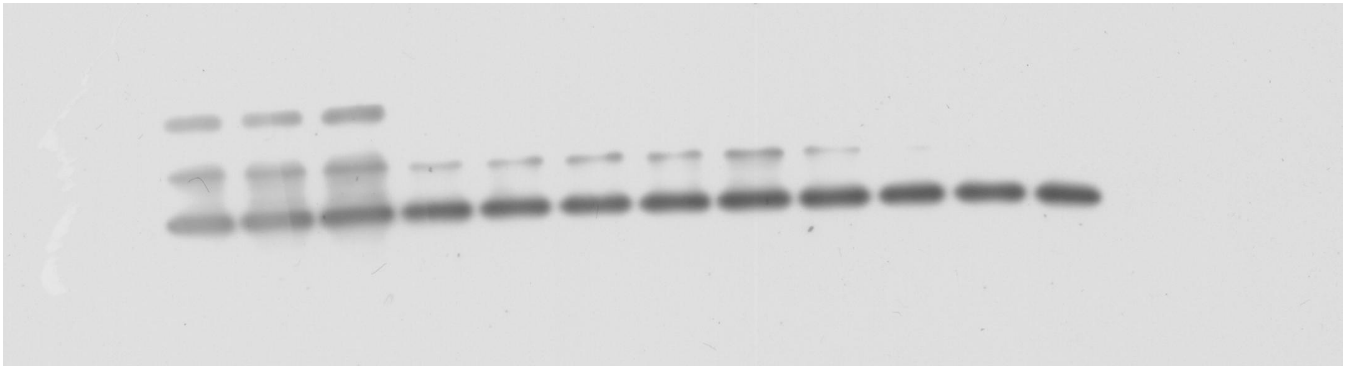

Figure S3

B

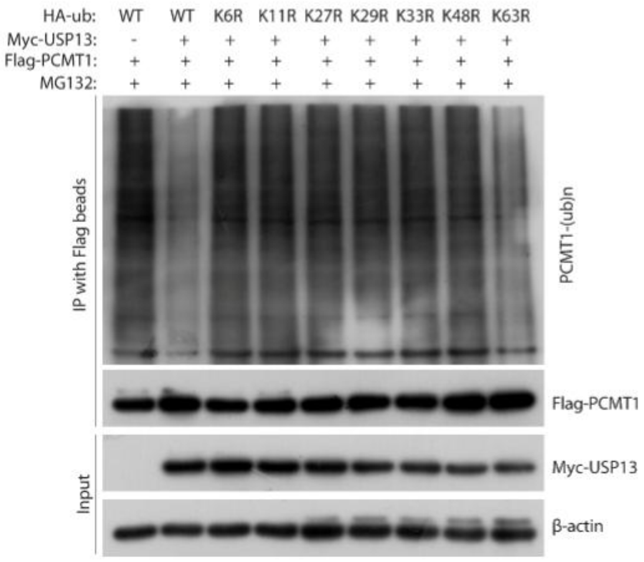
HA-ub: WT WT K6R K11R K27R K29R K33R K48R K63R
Myc-USP13: - + + + + + + + +
Flag-PCMT1: + + + + + + + + +
MG132: + + + + + + + + +
IP with Flag beads
PCMT1-(ub)n
Flag-PCMT1
Input
Myc-USP13
β-actin

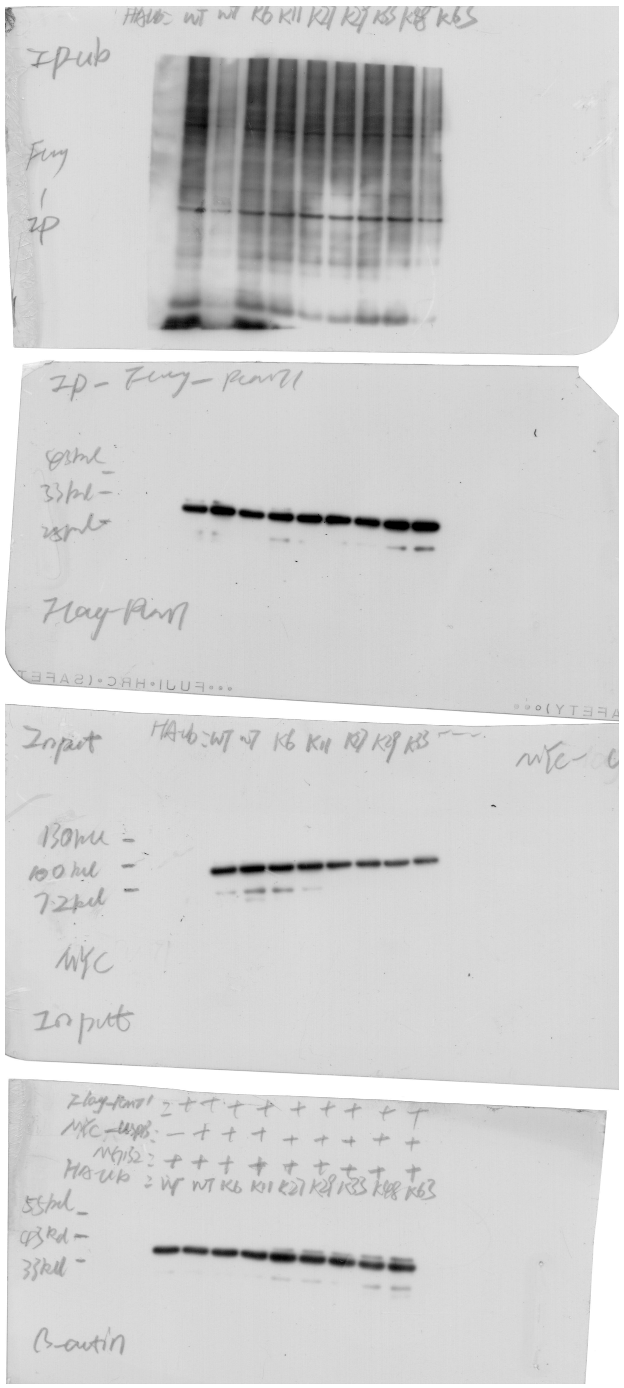

Figure S5

B

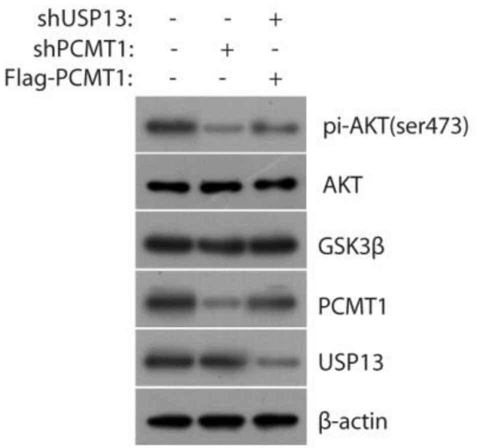
shUSP13: - - +
shPCMT1: - + -
Flag-PCMT1: - - +
pi-AKT(ser473)
AKT
GSK3β
PCMT1
USP13
β-actin

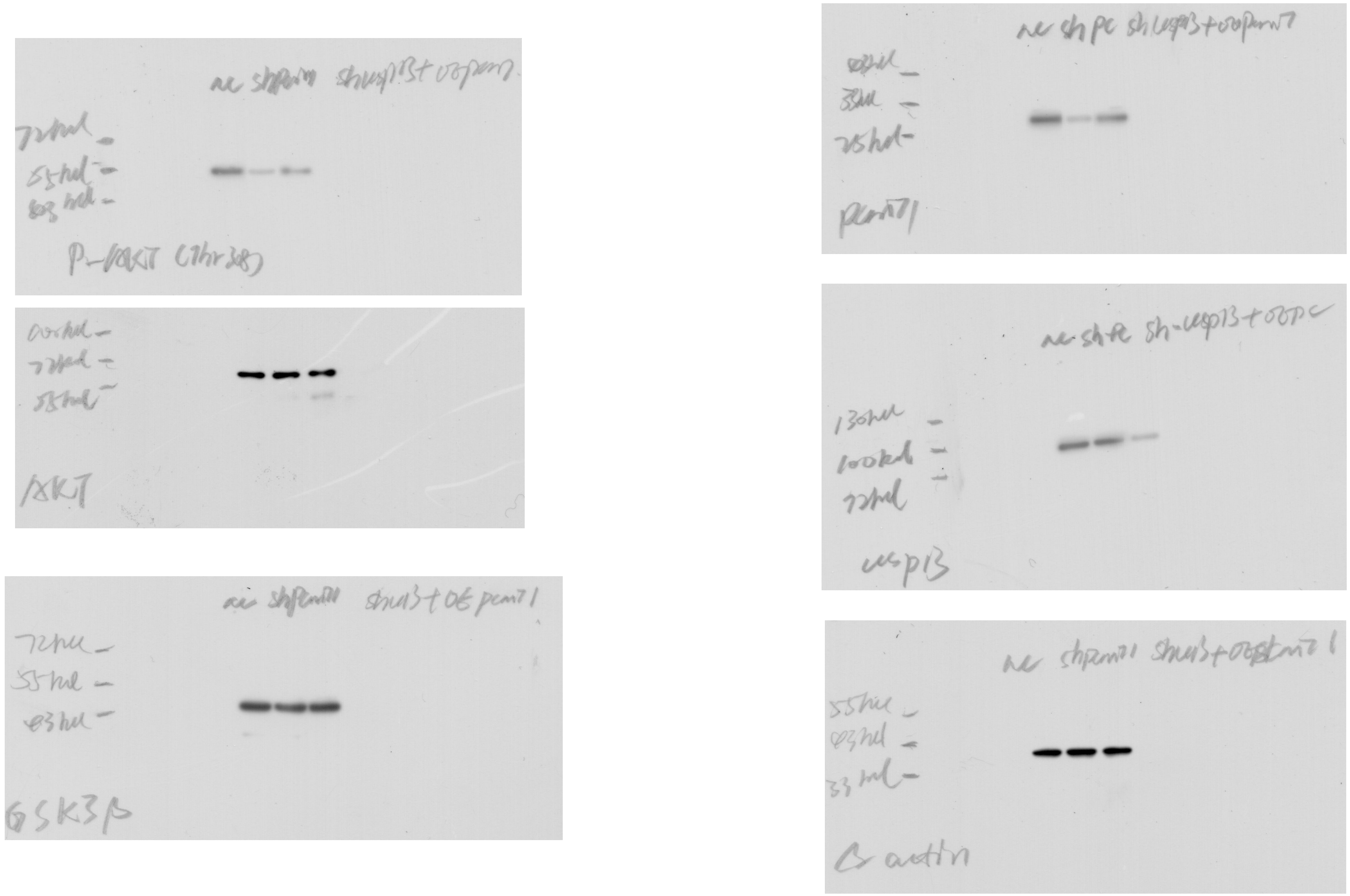

Figure S6

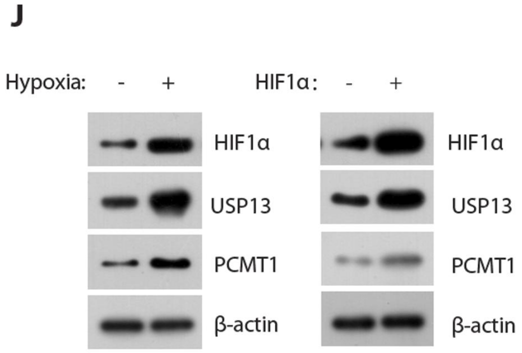
J
Hypoxia: - +
HIF1α : - +
HIF1α
USP13
PCMT1
β-actin
HIF1α
USP13
PCMT1
β-actin

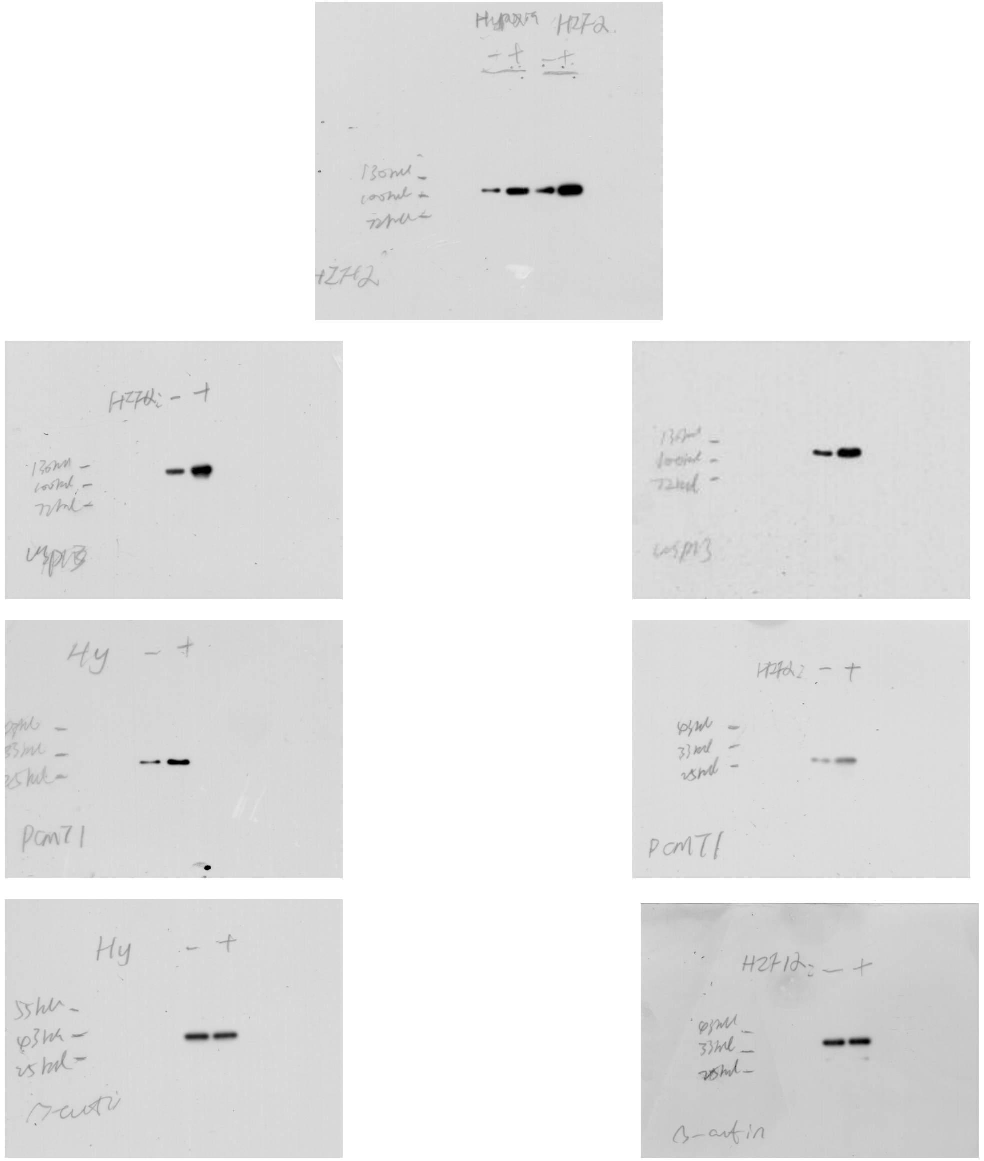

Figure S6

H

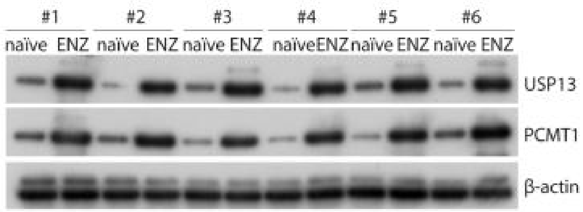

#1
#2
#3
#4
#5
#6
naïve ENZ naïve ENZ naïve ENZ naïveENZ naïve ENZ naïve ENZ
USP13
PCMT1
β-actin

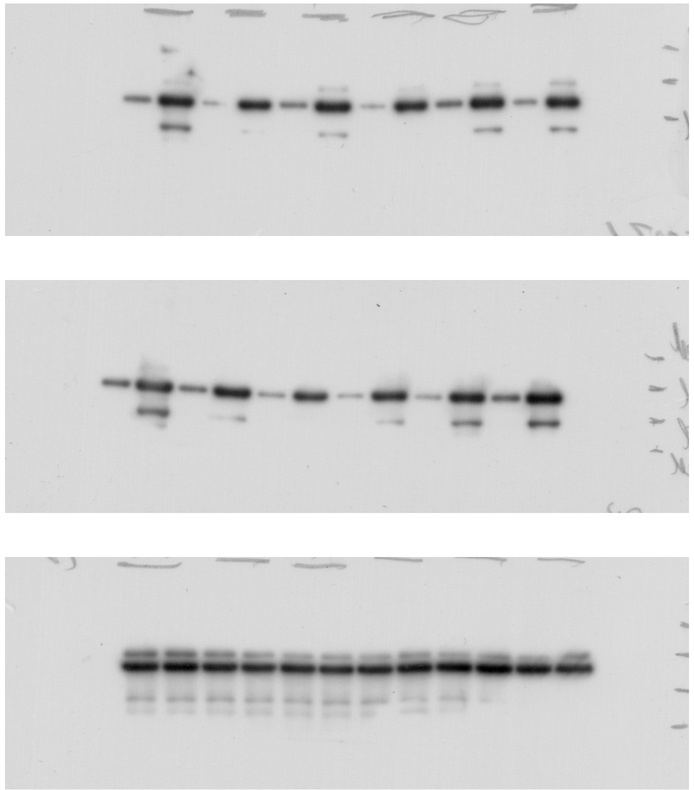

Figure S6

M

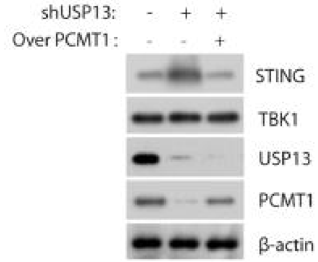
shUSP13: - + +
Over PCMT1 : - - +
STING
TBK1
USP13
PCMT1
β-actin

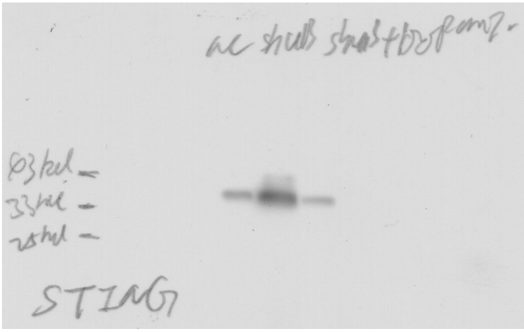
STING

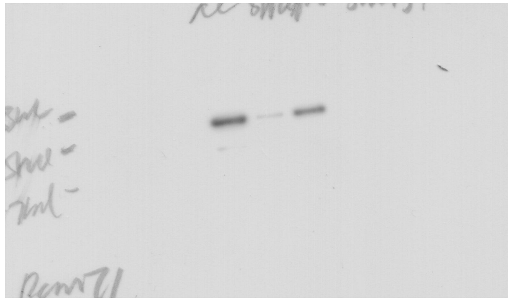

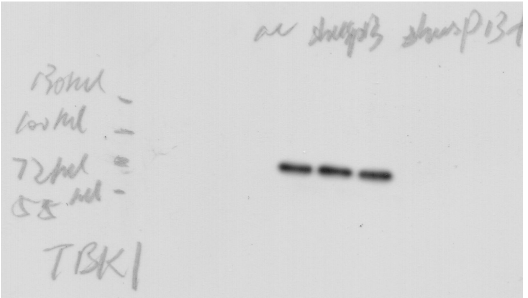
TBK1

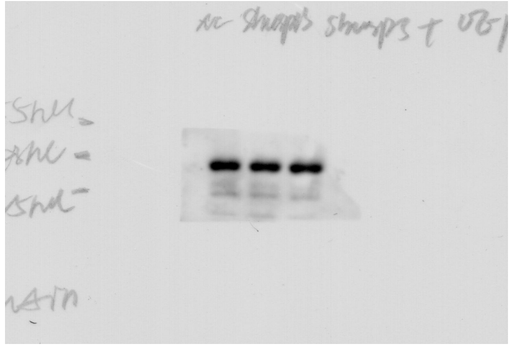

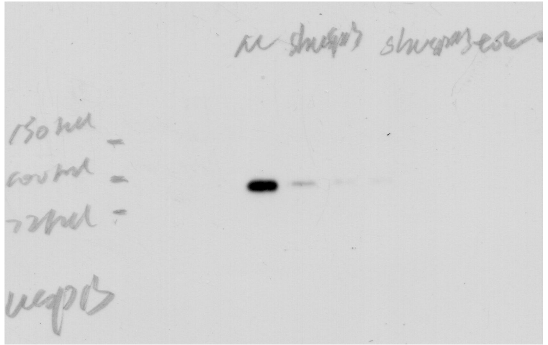

Supplement: Supplementary file 2 — original Western blots [file 41419_2026_8824_MOESM2_ESM.pdf]
